# Diaryl pyrimidine guanidine suppresses hepatocellular carcinoma cell stemness by targeting β-catenin signaling

**DOI:** 10.3389/fonc.2025.1641979

**Published:** 2025-09-01

**Authors:** Xuechun Chen, Dongxuan Ni, Jinghui Cheng, Bin Liang, Ruihan Zhang, Weilie Xiao, Rong Liu

**Affiliations:** ^1^ Translational Cancer Research Center, Peking University First Hospital, Beijing, China; ^2^ Key Laboratory of Medicinal Chemistry for Natural Resource of Ministry of Education, Yunnan Characteristic Plant Extraction Laboratory Co., Ltd., Yunnan Research and Development Center for Natural Products, School of Life Sciences and School of Pharmacy, Yunnan University, Kunming, China; ^3^ Southwest United Graduate School, Kunming, China

**Keywords:** diaryl pyrimidine guanidine, CSCs, HCC, β-catenin/TCF4, lenvatinib resistance, combination treatment

## Abstract

**Background:**

Liver cancer remains a major global health burden, with hepatocellular carcinoma (HCC) accounting for approximately 80% of liver cancer cases. Cancer stem cells (CSCs) play a critical role in HCC initiation, progression, metastasis, and resistance to therapy, making them critical targets for novel therapeutic interventions. However, effective agents specifically targeting CSCs in HCC remain limited. The objective of this study was to identify and characterize novel small molecules that inhibit CSCs properties and overcome drug resistance in HCC.

**Methods:**

Functional assays assessed the effects of C504244 on tumor sphere formation, cancer cell proliferation, and migration. RNA sequencing was conducted on C504244-treated HCC cells to investigate changes in gene expression profiles. Downstream targets of the Wnt signaling pathway were analyzed to determine pathway inhibition. Co-immunoprecipitation (Co-IP) was performed to assess whether C504244 disrupts the interaction between β-catenin and Transcription Factor 4 (TCF4) in HCC cells. Lenvatinib-resistant HCC cell lines were used to evaluate the combinatorial efficacy of C504244 and Lenvatinib *in vitro* and *in vivo*.

**Results:**

C504244 significantly suppressed tumor sphere formation, proliferation, and migration of HCC cells. Transcriptome analysis revealed that C504244 treatment led to significant inhibition of the Wnt signaling pathway, with corresponding downregulation of downstream target gene expression. Mechanistically, C504244 disrupted the β-catenin/TCF4 complex formation, which may contribute to reduced transcriptional activity. Since β-catenin signaling is hyperactivated in Lenvatinib-resistant HCC cells, C504244 was tested in combination with Lenvatinib and found to markedly sensitize these resistant cells to Lenvatinib treatment both *in vitro* and *in vivo*.

**Conclusions:**

C504244 represents a promising agent that effectively inhibits β-catenin signaling, thereby impairing CSCs properties and reversing Lenvatinib resistance in HCC cells. These findings suggest that C504244 may serve as a potential therapeutic agent for HCC.

## Introduction

HCC is one of the leading causes of cancer-related deaths worldwide, primarily due to late-stage diagnosis, metastasis, and the development of resistance to available therapies (1, 2). Treatment options available for early-stage HCC patients usually include surgical resection, liver transplantation, and radiofrequency ablation. However, in advanced-stage HCC patients, who are no longer eligible for resection interventions, systemic therapies, such as chemotherapy and target therapy, are the only treatment option that can benefit them (3, 4). Recently, targeted therapies such as tyrosine kinase inhibitors (TKIs) have become a major focus of clinical treatment for HCC (4–6). Lenvatinib, a multi-targeted TKI, is one of the approved and most effective first-line treatments for advanced HCC. It is able to target tyrosine kinases, such as vascular endothelial growth factor receptors (VEGFR), fibroblast growth factor receptors (FGFR), and platelet-derived growth factor receptors (PDGFR), KIT, and RET to inhibit tumor angiogenesis and growth (5, 7). Although Lenvatinib has shown promising effects in improving progression-free survival of HCC patients, the development of drug resistance remains a significant challenge. Preclinical *in vitro* and *in vivo* studies indicate that TKIs may have off-target effects, which might also contribute to tumor recurrence and metastasis (8). Clinically, only approximately 30% of HCC patients initially respond to TKIs, and nearly all responders develop resistance within six months (8, 9). Therefore, new therapeutic strategies are needed to overcome this resistance and improve long-term outcomes for HCC patients.

CSCs have emerged as a critical factor in the progression and recurrence of various cancers, including HCC (10, 11). CSCs are a small subpopulation of tumor cells with the ability to self-renew, differentiate, and initiate tumors. These cells are often more resistant to conventional therapies, contributing to relapse and metastasis (12, 13). In HCC, CSCs are thought to be responsible for tumor initiation, progression, and resistance to both chemotherapy and targeted therapies (14). Therefore, targeting CSCs represents a promising strategy for improving the effectiveness of current treatments.

Several signaling pathways are well-known to regulate CSCs properties, including the Wnt/β-catenin pathway (15, 16). It has been addressed that aberrant activation of the Wnt/β-catenin signaling axis contributes to the maintenance of CSCs, therefore promotes cancer proliferation and survival (16, 17). β-catenin, the key effector of the Wnt pathway, is a central player in regulating CSCs functions, and its stabilization in the nucleus leads to the activation of target genes that promote tumorigenesis and CSCs maintenance (17–19). In HCC, the Wnt/β-catenin signaling pathway is frequently dysregulated and is associated with aggressive disease progression (19–21). Therefore, targeting this pathway has become a major focus in the development of novel CSCs-targeting strategies for HCC. Drugs that inhibit the Wnt pathway have shown promise in preclinical models, and several small molecules and biologics have entered clinical trials (22, 23). However, there is still no approved therapy specifically targeting CSCs in HCC, and challenges still remain in translating these findings into clinical practice.

The tumor sphere formation assay has been developed as an *in vitro* surrogate method to study CSCs potential (24, 25), we therefore screened a series of compounds in our in-house library using HCC sphere model to identify potential CSCs inhibitors. During 34 compounds examined, we identified C504244 as the most potent inhibitor of tumor sphere formation in HCC cell line Huh7. Further investigation revealed that C504244 effectively suppresses HCC CSCs proportion, as well as cancer cell proliferation and migration. Mechanism study revealed C504244 was able to efficiently disrupt β-catenin/TCF4 complex formation and suppress β-catenin downstream targets’ expression. Furthermore, we found that C504244 treatment could sensitize Lenvatinib-resistant HCC cells to Lenvatinib, suggesting C504244 could be a promising strategy to overcome Lenvatinib resistance. This discovery holds clinical potential, offering a new approach for HCC treatment.

## Materials and methods

### Cell lines

Huh7, SK-Hep1, Hep1–6 liver cancer cell lines were purchased from the American Type Culture Collection (ATCC) and authenticated by short tandem repeat (STR) profiling, which was performed by Qida Biotechnology (Shanghai, China). All liver cancer cell lines were cultured in Dulbecco’s Modified Eagle Medium (DMEM) (#10-013-CVRC, Corning, VA, United States) medium supplemented with 10% fetal bovine serum (FBS) (#10099-141, Gibco, NY, United States). All cells were maintained in a humidified incubator at 37°C with 5% CO_2_.

### Sphere formation assay

HCC cells were seeded in low-adhesion 96-well plate at a density of 1,000 cells/well, with fresh culture medium replenished every three days. 10 days after culture, tumor spheres with diameter greater than 100μm were counted under a microscope. F12/DMEM supplemented with 1×B27, 20 ng/mL 20 ng/mL epidermal growth factor (EGF), 10 ng/mL fibroblast growth factor 10 (FGF10), and 10 ng/mL hepatocyte growth factor (HGF) and 1% PS was used as culture medium for sphere formation.

### Aldehyde dehydrogenase analysis

The ALDEFLUOR™ assay kit (#01700, STEMCELL Technologies, Vancouver, BC) was used for ALDH activity detection following the manufacturer’s protocol. A total of 2×10^5^ cells were centrifuged at 250g for 5 minutes, and the supernatant was discarded. The pellet was washed twice with the assay buffer. Cells was resuspended in 400μL of assay buffer mixed with 3μL activated ALDEFLUOR reagent, followed by dividing into 2 equal parts. 1 part were added with 3μL N, N-Diethylaminobenzaldehyde (DEAB) inhibitor to serve as the negative control, and the other part as the experimental one. Cells were incubated in dark at 37°C for 45 minutes. After incubation, cells were centrifuged at 250 g for 5 minutes, washed twice with the assay buffer, and resuspended in 300μL assay buffer for flow cytometry analysis within 4 hours on (#CytoFLEX S, Beckman Coulter Inc, CA, United States).

### CD24 staining flow cytometry assay

2×10^5^ cells were centrifuged at 250 g for 5 minutes, and the supernatant was discarded. Cells were washed twice with staining/washing buffer (1×Phosphate-Buffered Saline (PBS) with 2% FBS). Each sample was then resuspended in 300μL of buffer and incubated with 5μL of CD24 antibody (#PMG555428, Becton, Dickinson and Company, NJ, United States) on ice for 25 minutes. After incubation, cells were centrifuged at 250 g for 5 minutes, washed twice, resuspended and filtered for flow cytometry analysis within 4 hours on (#CytoFLEX S, Beckman Coulter Inc, CA, United States).

### Western blot assays

Tumor cells were lysed using RIPA lysis buffer (#P0013B, Beyotime, Shanghai, China), and protein concentration was quantified using the BCA Protein Assay Kit (#A55865, Thermo Fisher Scientific, MA, United States). The lysates were then subjected to SDS-PAGE and transferred onto PVDF membranes (#ISEQ00010, Millipore, Boston, United States). The membranes were blocked with 5% BSA for 1 hour at room temperature and incubated with primary antibodies at 4°C overnight. Subsequently, the blots were incubated with the horseradish peroxidase conjugated secondary antibody and developed by enhanced chemiluminescence.

Primary antibodies used were listed as following: Nanog (#4903), OCT4 (#2750), Sox2 (#2738), Sox9 (#D8G9H), GAPDH (#14C10), p-β-catenin-34/37 (#9561), β-catenin (#9562), p-GSK-3β (#9336), CyclinD1 (#2922), and TCF4 (#2569) were purchased from CST (United States), c-Myc (#9E10) was from Santa Cruz (United States).

### Reverse transcription quantitative polymerase chain reaction assays

Total RNA was extracted using TRI-Reagent (#TR118, Molecular Research Center, Inc, Cincinnati, OH, United States). Gene expression levels were measured using SYBR Premix Ex Taq II (Takara Bio, Shiga, Japan) on a 7,300 Real-Time PCR system (Applied Biosystems, Franklin Lakes, NJ, United States) with designed primers for target genes. The primers used in this study are listed in **Supplementary Table S1**.

### Colony formation assay

Cells in the logarithmic growth phase were counted, and 1,000 cells were seeded into each well of a 6-well plate. The cells were cultured for 10–14 days, with fresh medium changed every 3days. Colonies were fixed with 1mL 4% paraformaldehyde for 15 minutes, followed by staining with 0.1% crystal violet at room temperature for 20 minutes. Subsequently, stained colonies were washed with water until no residual dye remained. After the plate dried, images were taken. Finally, 10% acetic acid solution was added to dissolve the crystal violet for absorbance measuring at 530 nm using a microplate reader (#Multiskan SkyHigh, Thermo Fisher Scientific, Massachusetts, United States).

### Cell migration and invasion assay

Cells were trypsinized, washed with PBS, resuspended in serum-free medium, and adjusted to a density of 2×10^5^/mL. 500 μL medium containing 20% FBS was added to the lower chamber, 100-200μL of cell suspension was added to the upper chamber, followed by incubation for desired time course. Afterward, the chambers were washed with PBS, and a cotton swab was used to remove non-migrated cells on the upper chamber side from the membrane. Migrated cells were fixed with 4% paraformaldehyde for 20 minutes, stained with 0.1% crystal violet for 30 minutes, and washed with PBS for three times. The chambers were dried at 37°C, and microscopic images were captured, with the number of migrated cells counted.

For the cell invasion assay, 20% Matrigel (#353097; Corning, NY, United States) diluted with serum-free medium was added to the upper chambers to mimic the extracellular matrix before the assay.

### Immunofluorescence assay

3.5×10^4^ cells were seeded onto coverslips in 12-well plates and cultured for 48–72 hours until an appropriate confluence was achieved. After washing with 1×PBS, the cells were fixed with 1mL 4% paraformaldehyde for 15 minutes. Next, the cells were permeabilized with 0.2% Triton X-100 for 10 minutes, followed by three washes with PBS. The cells were then blocked with 3% BSA for 15 minutes, followed by incubating with the primary antibody overnight at 4°C. Then, the cells were washed and incubated with Alexa Fluor 488-conjugated goat anti-rabbit IgG (H + L) (#ZF-0511, ZSGB-Bio, Beijing, China) for 1 hour in the dark, after washing, cells were mounted in DAPI-containing mounting medium (#P36941, Thermo Fisher Scientific, Massachusetts, United States) for imaging on a fluorescence microscope (FV3000, Olympus Corporation, Japan).

### Immunoprecipitation assay

The IP assay was performed using an IP kit (P2197M, Beyotime, Shanghai, China). First, 300 μL of IP lysis buffer and 40 μL of Protein A magnetic beads were added to a sterile, enzyme-free EP tube. The mixture was thoroughly mixed, and the beads were separated using a magnetic stand. After washing the beads with PBS, the supernatant was discarded. In the experimental group, 350 μL of diluted primary antibody was incubated with the magnetic beads, while the control group was incubated with IgG. The incubation was carried out at 4°C with rotation for 8 hours. After cell lysis, proteins were extracted using IP lysis buffer, and their concentrations were determined. Equal amounts of protein were incubated with the magnetic beads for 8 hours. The beads were washed 5–6 times to remove nonspecific proteins. After the final wash, the beads were transferred to a new microcentrifuge tube, and 40 μL of 1×loading buffer was added. The samples were heated at 100°C for 10 minutes to denature the proteins. The denatured samples were separated by SDS-PAGE and analyzed by Western blot to detect the expression of the target protein.

### Chromatin immunoprecipitation assays

ChIP was performed using the ChIP kit (#P2080S, Beyotime, Shanghai, China) according to the manufacturer’s manual. In brief, ells were crosslinked with 3.7% formaldehyde, and crosslinking was terminated with glycine. After washing with PBS, cells were lysed in SDS Lysis Buffer containing protease inhibitors and incubated on ice. Chromatin was then fragmented to 200–1000 bp by sonication, and the shearing efficiency was checked by agarose gel electrophoresis. After centrifugation to remove the pellet, the supernatant was collected and diluted with ChIP Dilution Buffer. The sample was incubated with antibody targeting designed antigen, followed by immunoprecipitation using Protein A/G magnetic beads to enrich DNA fragments bound to the target protein. The immunocomplexes underwent a series of stringent washing steps to remove nonspecific binding. Finally, the target DNA was eluted using elution buffer, and crosslinking was reversed under high-temperature conditions. The DNA was then extracted and analyzed by RT-qPCR. The primers used in this study are listed in **Supplementary Table S1**.

### Xenograft assays

The animal protocols were approved by the Biomedical Ethics Committee, Subcommittee on Laboratory Animal Welfare, Peking University (PUIRB-LA2022626). All mice were purchased from Vital River Laboratory Animal Technology Co. (Beijing, China) and were subcutaneously implanted with Hepa1–6 cells at a concentration of 5 × 10^5^ cells per site in 6-week-old BALB/c nude mice. Once tumors reached approximately 50 mm³ in volume, the mice were randomly divided into four groups (six mice per group) for drug administration. Tumor volume and body weight were measured daily throughout the treatment period. Tumor volume was calculated using the formula: volume (mm³) = L × W² × 0.5 (where L is the longest diameter and W is the shortest diameter). At the end of the treatment, mice were euthanized and tumors were harvested for further analysis.

### Data and code availability

RNA sequencing data have been deposited at Genome Sequence Archive for Human HRA006499 and are publicly available as of the date of this publication.

### Compound characterization

^1^H and ^13^C Nuclear Magnetic Resonance (NMR) spectra were recorded on a Bruker AM-400 MHz spectrometer using C_2_D_6_OS (Deuterated Dimethyl Sulfoxide (DMSO)-d_6_) as the solvent and tetramethyl silane (TMS) as the internal standard. Chemical shifts (δ) were reported in parts per million (ppm), and coupling constants (J) were expressed in hertz (Hz). NMR spectroscopy was used for structural elucidation of the compounds, and the detailed spectral data are shown in **Supplementary Table S2** and **Supplementary Figures S1A, B**.

High-resolution mass spectra were obtained using an Agilent Liquid Chromatography/Mass Selective Detector Time-of-Flight (LC/MSD TOF) mass spectrometer. The mass spectrometric analysis provided accurate molecular weight information, which was used to confirm the molecular formula of the isolated compounds. The detailed High-Resolution Electrospray Ionization Mass Spectrometry (HRESIMS) data are presented in **Supplementary Table S2** and **Supplementary Figure S1C**.

High-Performance Liquid Chromatography (HPLC) analysis was carried out using an Agilent 1260 instrument equipped with a Gemini-NX C18 110A column (4.6 × 250 mm, 5 μm). The elution was performed at a flow rate of 1 mL/min with a gradient from 5% mixed solvent (99.5% acetonitrile + 0.5% triethylamine in water) to 100% mixed solvent over 20 minutes, followed by 5 minutes at 100% mixed solvent. HPLC was used to assess the purity of the compounds, and the results are summarized in **Supplementary Table S3** and **Supplementary Figure S1D**.

### Statistical analysis

All experimental data were analyzed using GraphPad Prism 5 software. The results are presented as mean ± standard error of the mean (Mean ± SEM). For comparisons involving only two groups, a two-tailed Student’s t-test was used to calculate the p-value. When comparing more than two groups, one-way analysis of variance (One-Way ANOVA) was applied to calculate the p-value. A p-value less than 0.05 was considered statistically significant. The significant differences in the results are indicated with an asterisk: *p < 0.05.

## Results

### Identification and validation of C504244 as a CSCs-inhibitory compound

**Figure 1:**
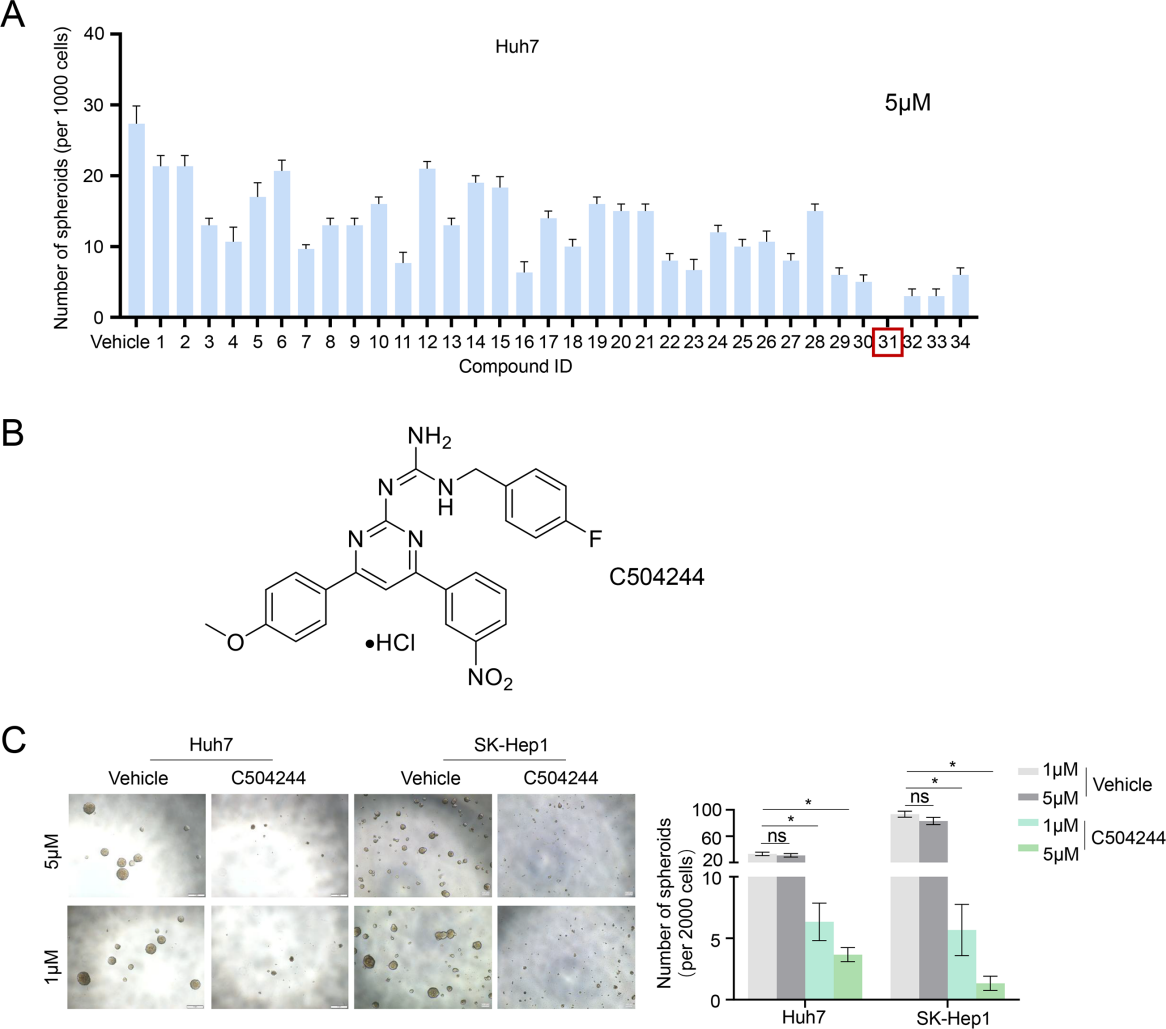
Identification of C504244 as a potent inhibitor of HCC sphere formation. **(A)** A panel of 34 small-molecule compounds from our in-house chemical library was screened in Huh7 spheres at a concentration of 5 μM. The number of tumor spheres with a diameter greater than 100 μm was counted. **(B)** The chemical structure of compound C504244. **(C)** Huh7 and SK-Hep1 cells were treated with vehicle Control (DMSO) or C504244 at indicated dosage for 6 days. Representative images of tumor spheres were captured, and the number of tumor spheres with a diameter greater than 100 μm was counted. All statistical analyses were performed using Student’s *t*-test with significance indicated as *p < 0.05.

To identify small molecules with potential inhibitory effects on CSCs stemness, we performed a primary screen using our in-house library containing 34 candidate compounds (5 μM) using sphere formation assay in Huh7 HCC cells. Among all compounds detected, compound 31 (C504244) exhibited the most potent suppression ability of sphere formation (**Figure 1A**; **Supplementary Figure S2**). Structurally, C504244 features a diaryl pyrimidine guanidine scaffold (**Figure 1B**). Its physicochemical properties were computationally evaluated using ADMETlab 3.0 (https://admetlab3.scbdd.com), and the results (**Supplementary Table S4**) indicate favorable ADMET parameters, supporting further investigation. To further validate the inhibitory effect of C504244 on CSCs stemness, we treated two HCC cell lines, Huh7 and SK-Hep1, with C504244 at indicated concentrations (**Figure 1C**). As the data shown in **Figure 1C**, C504244 exhibited strong suppression effects in a dosage-dependent manner in both cell lines, suggesting a robust and consistent inhibitory effect on CSCs properties. We further assessed the potential cytotoxicity of C504244 in four normal human cells, HUVEC (Human Umbilical Vein Endothelial Cells), HDF (Human Dermal Fibroblast), WI-38 (human embryonic lung fibroblast), and PBMC (Peripheral Blood Mononuclear Cell). As shown in **Supplementary Figure S3**, C504244 exhibited markedly lower toxicity in all four normal cells than in 2 HCC cells (Huh7 and SK-Hep1), supporting its tumor-selective activity.

### C504244 inhibits CSCs stemness in HCC cells

**Figure 2:**
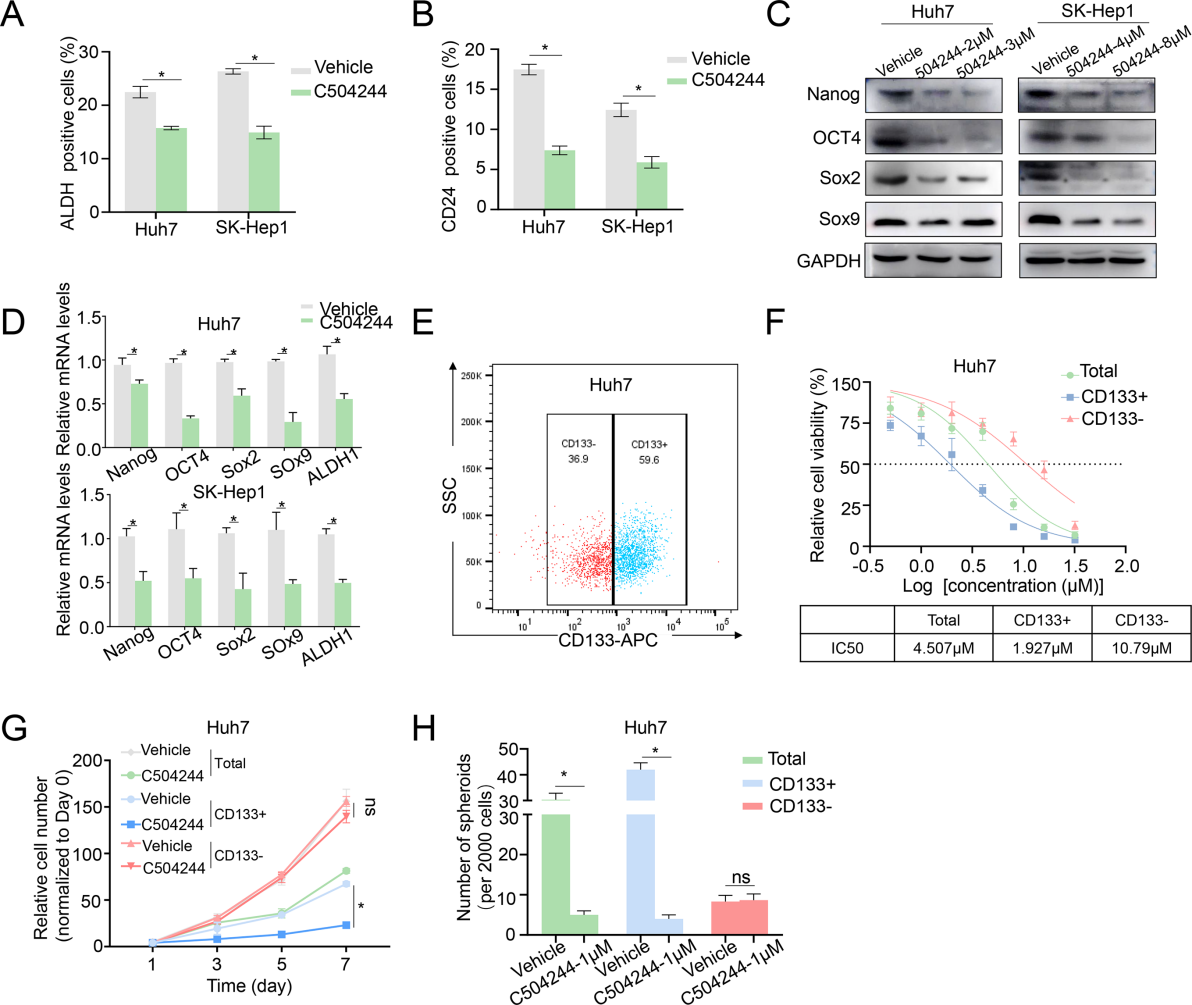
C504244 suppresses HCC CSCs maintenance. **(A-B)** DMSO or C504244 (2 µM) treated Huh7 and SK-Hep1 cells were collected for ALDH activity assay **(A)** and CD24 staining flow cytometry analysis**(B)**. The percentage of ALDH and CD24-positive cells was quantified in the bar graph. **(C-D)** Huh7 and SK-Hep1 cells treated with C504244 for 48 hours at indicated concentrations were collected for Western blot **(C)** and qPCRs **(D)** analysis. **(E)** Flow cytometric sorting of Huh7 cells to isolate CD133^+^ and CD133^-^ subpopulations. **(F)** Dose-response curves and IC50 values of C504244 in total, CD133^+^, and CD133^-^ Huh7 cells. **(G)** Huh7 cells plated in 96-well plates were treated with DMSO or C504244 at the day after seeding, cell numbers were counted every two days and monitored until day 7. Relative cell growth was normalized to day 1. **(H)** Huh7 cells were treated with vehicle Control (DMSO) or C504244 at indicated dosage for 6 days. Representative images of tumor spheres were captured, and the number of tumor spheres with a diameter greater than 100 μm was counted. All statistical analyses were performed using Student’s *t*-test with significance indicated as *p < 0.05, ns, no statistical significance.

We ALDH activity assay, CSCs marker CD24 flow cytometry analysis, and western blotting/qPCR analysis of CSCs markers, to further validate the inhibitory effect of C504244 on CSCs stemness. The results of both Huh7 and SK-Hep1 cells showed similar trends, with C504244 significantly reducing CSCs characteristics. As the data shown in **Figure 2**, compared to the control group, C504244 treatments significantly decreased the proportion of ALDH+ and CD24+ cells, further confirming its inhibitory effect on CSCs characteristics. Meanwhile, the expression of several well-known CSCs markers, such as Nanog, OCT4, Sox2, and Sox9, were noticeably suppressed in C504244 treated HCC cells at both protein and mRNA levels (**Figures 2C, D**). Additionally, C504244 also suppressed the mRNA expression of ALDH (**Figure 2D**), which might contribute to the reduced activity of ALDH in C504244-treated cells.

To further evaluate the CSC-targeting effects of C504244, we sorted Huh7 cells into CD133^+^ (HCC stem cells) and CD133^-^ (non-stem cells) subpopulations (26–28) and treated them with vehicle control or C504244 at indicated dosages. As shown in **Figures 2E–H**, CD133^+^ cells were markedly more sensitive to C504244 treatment, with an IC50 of 1.927 μM, compared to 10.79 μM in CD133^-^ cells (**Figure 2F**). Consistently, C504244 inhibited CD133^+^ cell growth more severely than CD133^-^ cells (**Figure 2G**). Also, sphere formation ability was significantly reduced in CD133^+^ populations upon C504244 treatment, with no significant effects in CD133^-^ cells (**Figure 2H**). Taken together, these results further implicated the selective inhibitory effects of C504244 on CSC-like subpopulations in HCC.

### C504244 suppresses HCC cell growth and migration abilities

**Figure 3:**
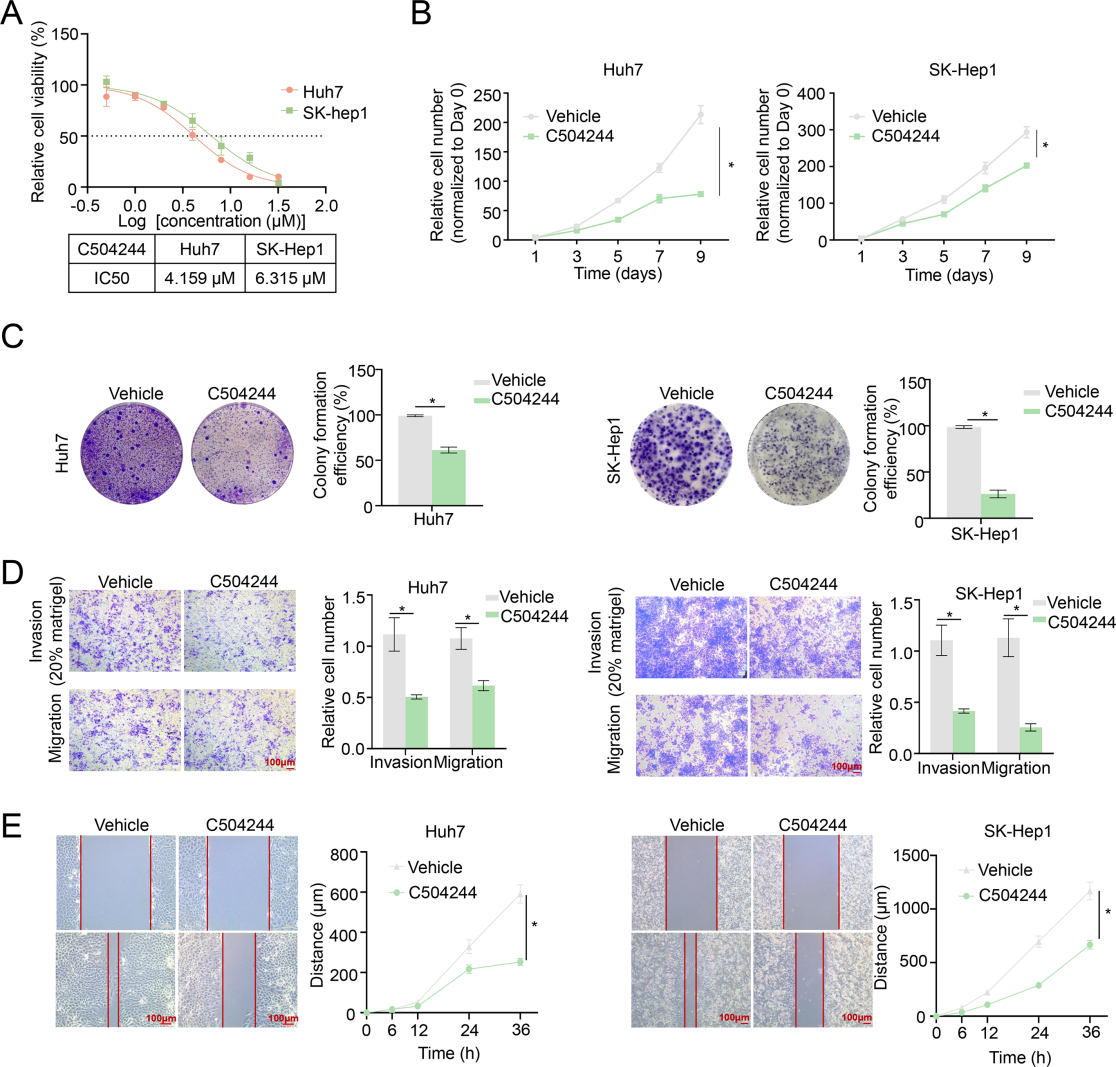
C504244 inhibits HCC cell proliferation and migration. **(A)** The IC_50_ values of C504244 in HCC cells were determined using cell viability assay. Huh7 and SK-Hep1 cells were treated with C504244 at indicated concentrations for 48 hours, followed by CCK-8 cell viability assay. **(B)** Huh7 and SK-Hep1 cells plated in 96-well plates were treated with DMSO or C504244 at the day after seeding, cell numbers were counted every two days and monitored until day 9. Relative cell growth was normalized to day 1. **(C)** Huh7 and SK-Hep1cells plated in 6-well plates were treated with DMSO or C504244 at the day after seeding. 14 days after drug treatment, the number of colonies formed was quantified, and representative images are shown. **(D)** DMSO or C504244 treated Huh7 and SK-Hep1 cells were collected for transwell migration and invasion assays. For invasion assays, 20% Matrigel was added to the transwell inserts. Cells that migrated or invaded through the membrane were stained with crystal violet and quantified by counting the number of cells. Scale bar = 100 µm. **(E)** Huh7 and SK-Hep1 cells treated with DMSO or C504244 for 24 hours were scratched for wound healing assay. Images were captured at 0- and 24-hours post-treatment. Scale bar = 100 µm. All statistical analyses were performed using Student’s *t*-test, with significance indicated as *p < 0.05.

In order to detected the effects of C504244 on HCC malignant progression, we first checked the role of it on cell viability, and found C504244 suppressed Huh7 and SK-Hep1 cell survival with IC_50_ as 4.159 µM and 6.315 µM, respectively (**Figure 3A**). Since both decreased cell growth and increased cell death contribute to suppressed cell viability, we first evaluated the effect of C504244 on cell proliferation using both growth curve analysis and colony formation assays. As shown in **Figures 3B, C**, treatment with C504244 significantly inhibited cell proliferation and colony formation in both Huh7 and SK-Hep1 cells, compared to the control group, confirming its inhibitory effect on HCC cell growth. We also checked cell apoptosis using Annexin V flow cytometry analysis, and found C504244 did not induce HCC cell apoptosis significantly (data not shown), indicating C50244 induced cell loss might predominantly cause by cell proliferation inhibition. We also examined the effects of C504244 on cell migration and invasion, the key characteristics of malignant progression. Transwell migration and Matrigel invasion assays (**Figure 3D**) revealed C504244 treatment significantly reduced both migration and invasion abilities of Huh7 and SK-Hep1 cells. Additionally, the wound healing assay (**Figure 3E**) showed impaired wound closure in C504244 treated cells, indicating slowed migration. In summary, C504244 effectively inhibits HCC cell proliferation, migration, and invasion, highlighting its potential as a therapeutic agent for targeting HCC progression.

### C504244 suppresses Wnt signaling by disrupting β-catenin/TCF4 interaction

**Figure 4:**
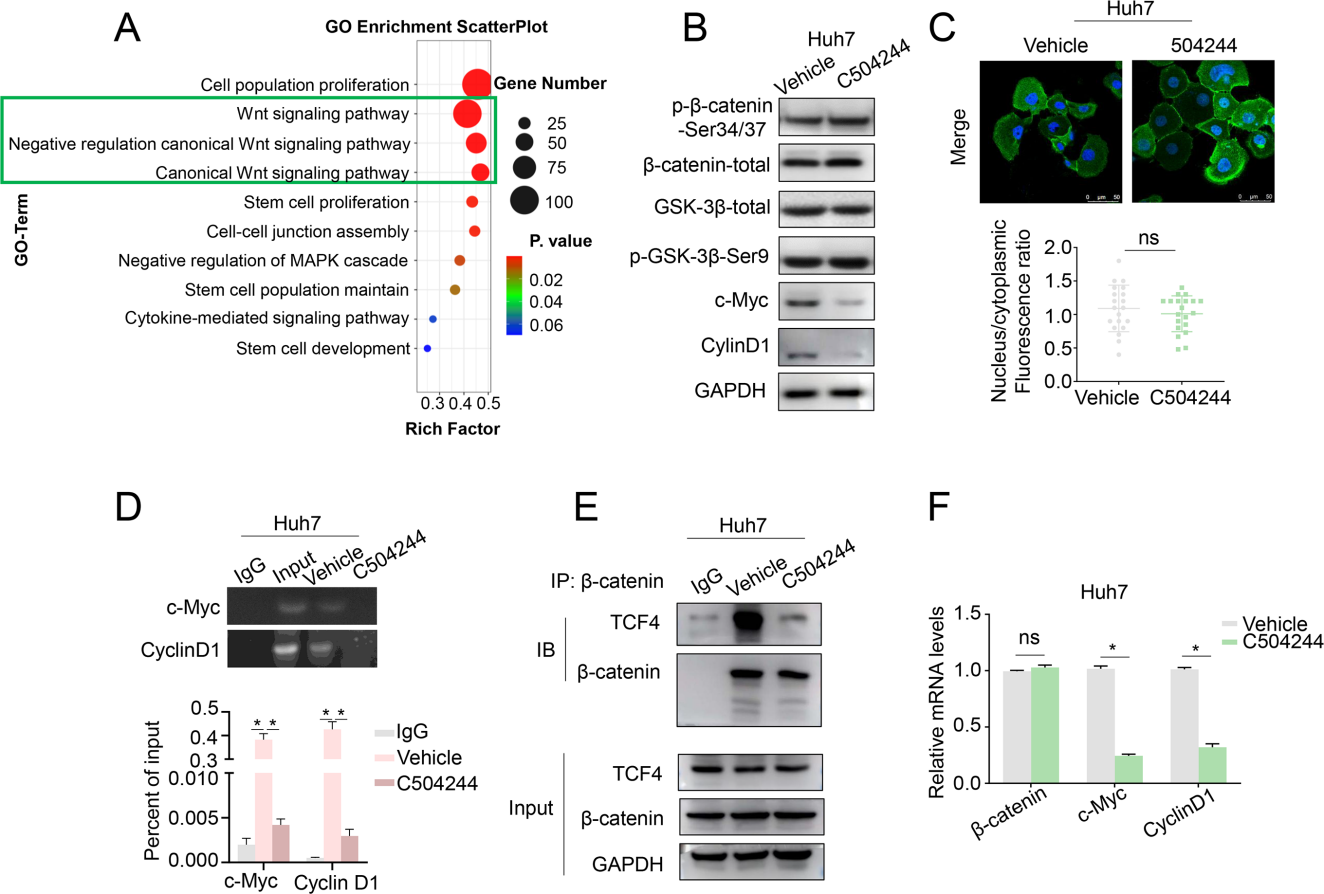
C504244 inhibits β-Catenin/TCF4 interaction. **(A)** GO analysis of C504244 treated compared to vehicle control Huh7 cell RNA sequencing data showing enrichment of the Wnt signaling pathway. **(B)** C504244 or DMSO treated Huh7 cells were collected for western blotting analysis, GAPDH was detected as the loading control. **(C)** Confocal immunofluorescence analysis of β-catenin in Huh7 cells treated with DMSO or C504244. The nucleocytoplasmic fluorescence ratio was quantified using ImageJ. Scale bar = 50 μm. **(D)** Binding of TCF4 to the promoter region of Cyclin D1 and c-Myc promoter was detected using chromatin immunoprecipitation (ChIP) assay in vehicle or C504244-treated Huh7 cells. **(E)** Co-immunoprecipitation (IP) analysis was performed to detect the interaction between TCF4 and β-catenin in vehicle or C504244-treated Huh7 cells. **(F)** mRNA expression levels of stemness-related genes were measured by qPCR in Huh7 cells treated with vehicle or C504244 (2 µM) for 48 hours. All statistical analyses were performed using Student’s *t*-test, with significance indicated as *p < 0.05, ns, no statistical significance.

To elucidate the molecular mechanisms underlying the effects of C504244 in suppressing CSCs maintenance and malignant progression in HCC, we performed RNA sequencing using C504244-treated Huh7 cells, followed by Gene Ontology (GO) analysis. Among all the biological pathways affected by C504244, the Wnt signaling pathway (**Figure 4A**) particularly attracted our attention because the Wnt/β-catenin pathway is a central regulator of CSCs self-renewal and malignant progression in HCC. Activation of this pathway stabilizes β-catenin, facilitating its nuclear translocation and interaction with TCF4, which drives the transcription of downstream targets such as Cyclin D1 and c-Myc (17, 18, 29). To validate the sequencing results, we examined several downstream target genes of the Wnt pathway. Consistent with the RNA sequencing data, treatment with C504244 significantly inhibited the expression of 2 classic targets of Wnt signaling, c-Myc and CyclinD1 (**Figure 4B**). Unexpectedly, C504244 treatments did not alter total β-catenin levels or its phosphorylation at Ser33/37/Thr41 (which is targeted by GSK-3β for proteasomal degradation) (**Figure 4B**), nor the phosphorylation of GSK-3β (Ser9), a key kinase regulating β-catenin stability (**Figure 4B**), ruling out the possibility of upstream kinase modulation. Moreover, immunofluorescence staining confirmed that the nuclear-cytoplasmic distribution of β-catenin was unchanged by C504244 treatments (**Figure 4C**). Given that the stability and localization of β-catenin were unaltered, we hypothesized that C504244 might interfere with its transcriptional activity. To test this possibility, we performed ChIP-qPCR to assess the binding of β-catenin/TCF complex to the promoters of its target genes. C504244 treatment led to a marked reduction in TCF4 occupancy at the c-Myc and Cyclin D1 promoter regions (**Figure 4D**), suggesting transcriptional repression. We further demonstrated that C504244 significantly impaired the formation of the β-catenin/TCF4 complex (**Figure 4E**), indicating that such compound disrupts their physical interaction. To further validate the inhibitory effect of C504244 on Wnt/β-catenin transcriptional activity, we performed qRT-PCR to assess the expression levels of key target genes. Consistent with previous results, treatment with C504244 significantly decreased c-Myc and Cyclin D1 mRNA expression (**Figure 4F**). These data provide evidences suggesting that C504244 represses Wnt signaling likely through inhibiting β-catenin/TCF4 interaction, thereby impairing the transcription of key oncogenic targets critical for HCC progression.

To determine whether the anti-tumor effects of C504244 are mainly dependent on β-catenin signaling or not, we checked the effects of C504244 on HCC cells under β-catenin knockdown condition in Huh7 cells. In consistent with our previous results, we found C504244 alone significantly reduced the ALDH+ cell population, while β-catenin depletion (**Supplementary Figure S4A**) could slightly further enhance this reduction (**Supplementary Figure S4B**). We also found C504244 treatment markedly inhibited cell proliferation and migration, while β-catenin knockdown did not further suppress these phenotypes in HCC cells (**Supplementary Figures S4C, D**), indicating β-catenin plays vital roles in mediating C504244’s functions in HCC cells.

### C504244 synergizes with lenvatinib to overcome resistance in HCC

Multiple studies have confirmed that the Wnt/β-catenin signaling pathway is frequently aberrantly activated in HCC, contributing to disease progression and therapeutic resistance through various mechanisms (30–32). One critical mechanism is its role in maintaining CSCs stemness, which drives tumor resistance to anti-cancer therapies (22, 23). Consistently, analysis of our HCC organoids (with paired clinical samples) database (33) revealed significantly higher Wnt signaling activity in tumor tissues/organoids compared to adjacent normal liver tissues/organoids (**Supplementary Figure S5A**). Moreover, Wnt signaling is positively correlated with CSCs characteristics in HCC organoids (**Supplementary Figure S5B**), reinforcing its role in sustaining cancer stemness.

**Figure 5:**
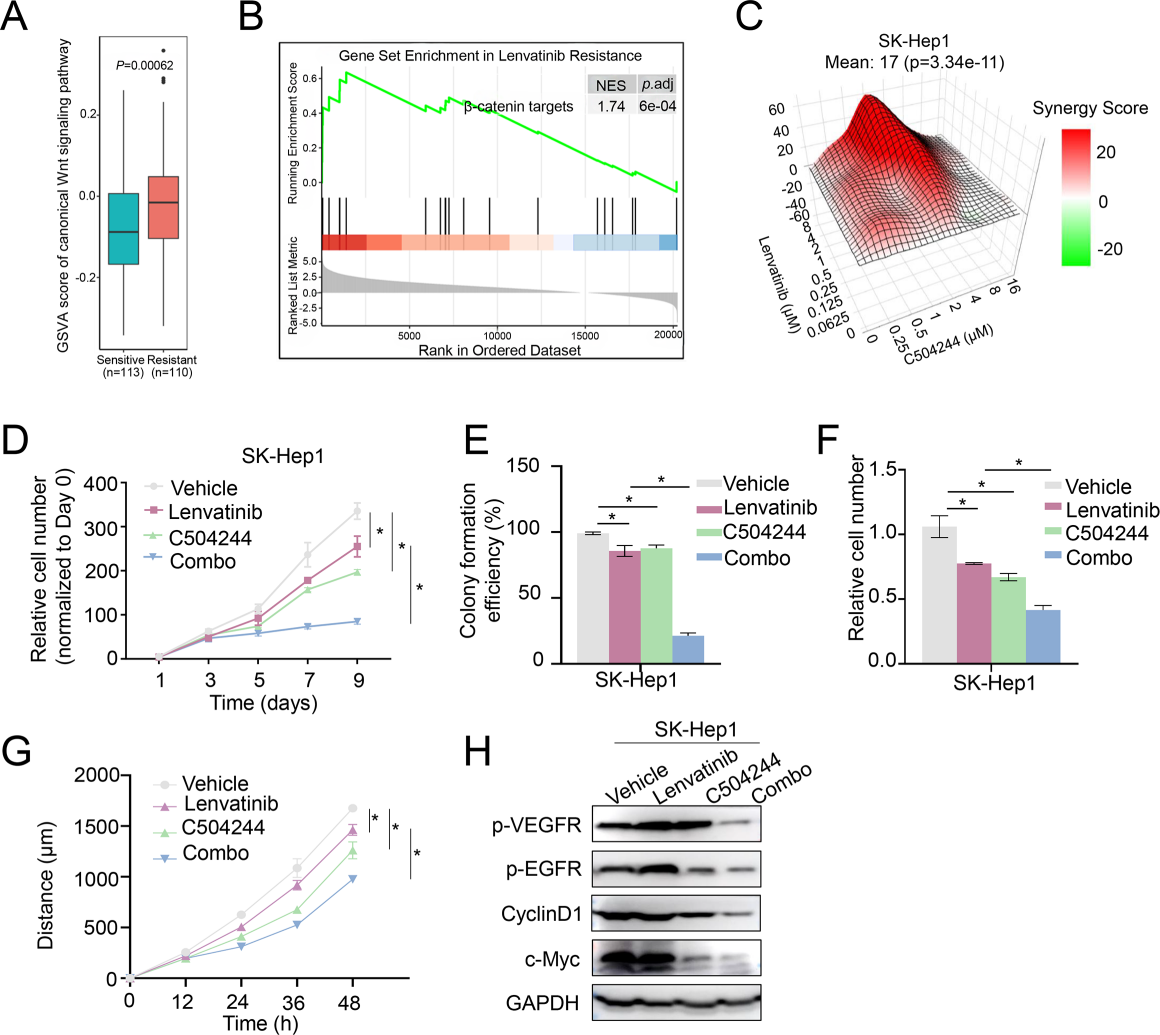
C504244 sensitize Lenvatinib resistant HCC cell to Lenvatinib. **(A)** GSVA of the canonical Wnt signaling pathway in Lenvatinib-sensitive (n=113) and Lenvatinib-resistant (n=110) organoids. **(B)** Gene Set Enrichment Analysis (GSEA) for β-catenin target genes in Lenvatinib-resistant organoids. The enrichment plot indicates significant upregulation of β-catenin target genes in resistant organoids. **(C)** Synergy map of SK-Hep1 cells treated with C504244 and Lenvatinib at indicated concentrations. **(D)** Cell proliferation was assessed by cell growth curve analysis of SK-Hep1 cells treated with DMSO, Lenvatinib (2 µM), C504244 (3 µM), or combination of both drugs. Cell numbers were counted every two days and monitored until day 9. Relative cell growth was normalized to day 0. **(E)** Colony formation assay was performed using SK-Hep1 cells treated with DMSO, Lenvatinib (2 µM), C504244 (3 µM), or combination of both drugs for 14 days. Colony formation efficiency was calculated by comparing colony numbers relative to the vehicle control. **(F)** SK-Hep1 cells treated with DMSO, Lenvatinib, C504244, or the combination for 24 hours were applied for cell migration assay. Scale bar = 100 µm. **(G)** SK-Hep1 cells treated with DMSO, Lenvatinib (2 µM), C504244 (3 µM), or the combination of both drugs were utilized for wound healing assay. Images were taken at 0- and 24-hours post-treatment. **(H)** SK-Hep1 cells treated with DMSO, Lenvatinib or C504244 were collected for western blotting analysis. GAPDH was detected as loading control. All statistical analyses were performed using Student’s *t*-test, with significance indicated as *p < 0.05.

CSCs have been represented as the major source of therapy resistance (10, 12). Resistance to Lenvatinib, a first-line treatment for advanced HCC, has severely restricted the clinical benefits of this drug. Utilizing our HCC organoids drug-sensitivity database (33), we analyzed GSVA (Gene Set Variation Analysis) score of Wnt signaling in Lenvatinib resistant organoids compared to sensitive ones, the results revealed significantly higher Wnt pathway activation in the resistant organoids (**Figure 5A**). Furthermore, GSEA (Gene Set Enrichment Analysis) confirmed the enrichment of β-catenin target genes in the resistant organoids (**Figure 5B**). These findings suggest that aberrant Wnt activation may contribute to the development of Lenvatinib resistance.

Given these findings, we hypothesized that inhibiting Wnt/β-catenin signaling pathway could enhance the therapeutic efficacy of Lenvatinib. To test this possibility, we utilized Lenvatinib-resistant SK-Hep1 cells to assess whether combining C504244 with Lenvatinib could improve treatment response (8, 34, 35). Indeed, C504244 treatment significantly sensitize SK-Hep1 cells to Lenvatinib, with the synergy score (calculated using Synergy Finder 2.0) of 17 (**Figure 5C**), indicating a strong synergistic effect. Cell growth curve and colony formation assays confirmed the synergistic effects of C504244 and Lenvatinib (**Figures 5D, E**; **Supplementary Figure S6A**). Similarly, migration and wound healing assays showed that the combination treatment effectively suppressed cell migration (**Figures 5F, G**; **Supplementary Figures S6B, C**), indicating that C504244 enhances the sensitivity of Lenvatinib-resistant cells to Lenvatinib. We confirmed the effects of C504244 and Lenvatinib on HCC cells by checking the activation status of their target signaling pathways, including phosphorylation of VEGFR/Epidermal Growth Factor Receptor (EGFR) and expression of c-Myc and Cyclin D1.

Lenvatinib inhibits VEGFR/EGFR signaling in Lenvatinib sensitive Huh7 cells as expected, and C504244 suppresses β-catenin signaling, thus combined application of both drugs blocks both VEGFR/EGFR and β-catenin signaling, which contributes the synergistic effects of these drugs in Huh7 cells (**Supplementary Figure S7**). While in **Figure 5H**, Lenvatinib failed to inhibit VEGFR/EGFR signalings in Lenvatinib resistant HCC cells, while C504244 suppresses β-catenin signaling, which meanwhile contributes to decreased EGFR activation (36), which might explain the synergistic effects of these drugs in HCC cells.

### Combined lenvatinib and C504244 treatment inhibits tumor growth *in vivo*

To further confirm the synergistic anti-tumor effect of Lenvatinib and C504244, we employed the Hep1–6 cell line, which is also resistant to Lenvatinib (35). Similar to the results observed in SK-Hep1 cells, the combination of Lenvatinib and C504244 exhibited a strong synergistic effect in Hep1–6 cells (**Supplementary Figure S8A**). Consistently, the combination treatment also significantly inhibited Hep1–6 cell proliferation, colony formation, and migration compared to either treatment alone (**Supplementary Figures S8B-D**).

**Figure 6:**
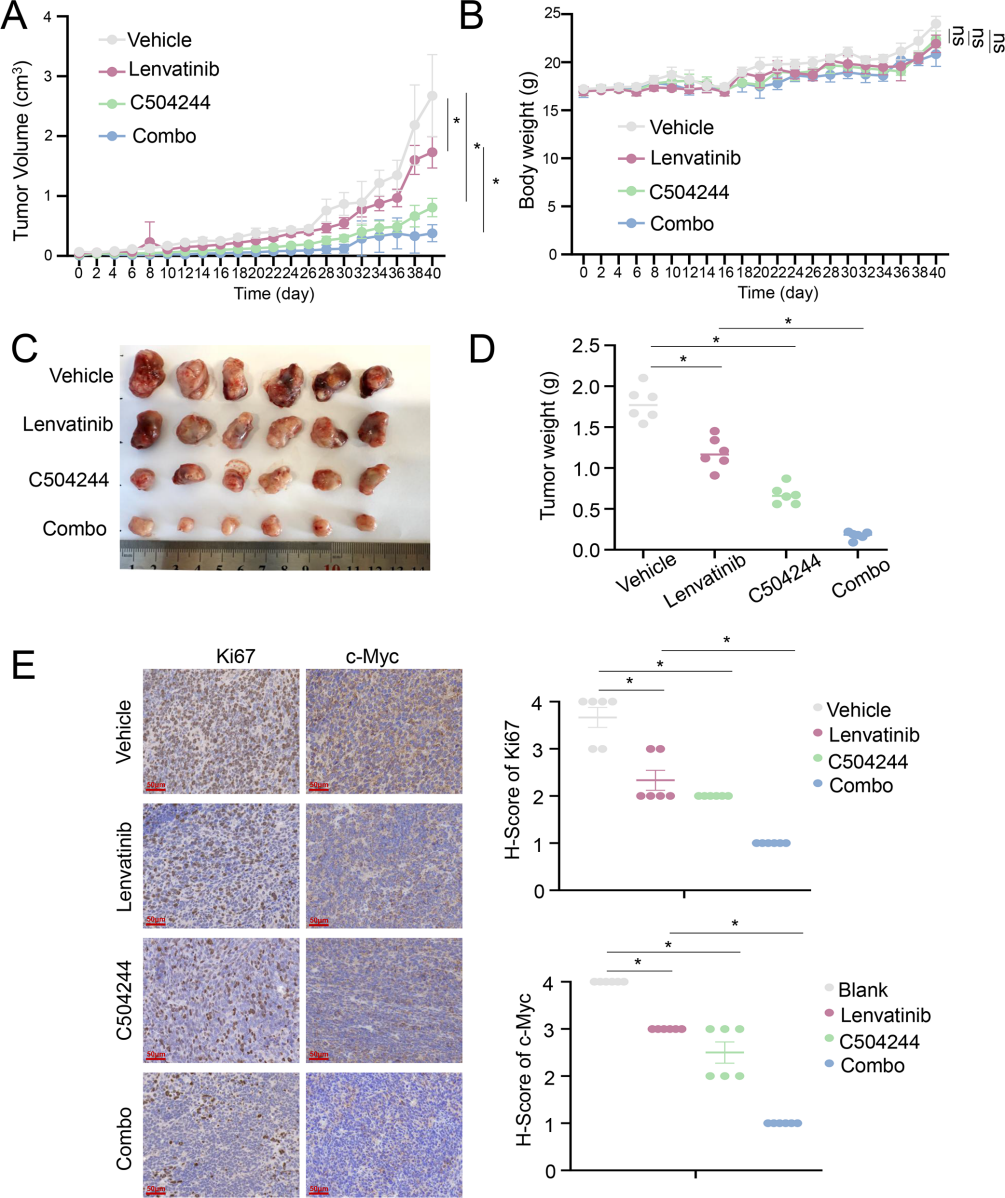
C504244 sensitize Lenvatinib resistant HCC cell to Lenvatinib *in vivo*. **(A-B)** Tumor growth curve of Hepa1–6 cells in nude mice treated with DMSO, Lenvatinib (4 mg/kg), C504244 (25 mg/kg), or the combination of both drugs. Tumor volume **(A)** and body weight **(B)** were measured daily, with every-other-day data displayed in the graphs for clarity. **(C-D)** Tumors excised at the end of the experiment **(C)** were weighed **(D)**. **(E)** Tumors from each group mentioned above were fixed and sectioned for IHC staining of Ki67 and c-Myc. Statistical significance was assessed by Student’s *t*-test, with significance indicated as *p < 0.05.

To assess the therapeutic potential of combining Lenvatinib and C504244 *in vivo*, nude mice were implanted with Lenvatinib-resistant murine Hep1–6 tumor cells and treated with DMSO, Lenvatinib (4 mg/kg), C504244 (25 mg/kg), or the combination of both agents (35). Tumor volume was monitored over the treatment period, and the results demonstrated a significant reduction in tumor growth in the combination treatment group compared to the single-agent treatment groups (**Figure 6A**), without affecting the body weight, indicating that combination of Lenvatinib and C504244 did not cause obvious toxicity (**Figure 6B**). Moreover, Hematoxylin and Eosin (H&E) staining of major organs (heart, liver, kidney, spleen, and lung) showed no evident tissue damage, inflammation, or necrosis in mice treated with C504244 alone or in combination with Lenvatinib, further supporting the safety of C504244 and the combination regimen at the tested doses (**Supplementary Figure S9**). Tumor masses were weighed at the end of the treatment period, and the data revealed a significant decrease in tumor weight in the combination group compared to the individual treatment groups (**Figures 6C, D**). Meanwhile, we noticed that the Ki67 and c-Myc positive cells were severely reduced in the combination group, which further confirmed C504244, in combination with Lenvatinib, exhibits synergistic effects and can reverse Lenvatinib resistance in liver cancer cells. These results collectively demonstrate that the combination of Lenvatinib and C504244 effectively inhibits tumor growth and reduces tumor weight *in vivo*, supporting the potential of this combination therapy for enhanced anti-tumor efficacy.

## Discussion

HCC remains a global health challenge worldwide, with late-stage diagnosis, aggressive metastasis, and therapeutic resistance significantly limiting patient survival benefits (1, 37). According to the 2024 American Society of Clinical Oncology (ASCO) guidelines, TKIs such as Lenvatinib or sorafenib remain key components of first-line therapy, typically in combination with immune checkpoint inhibitors (ICIs), such as PD-1/PD-L1 antibodies (4, 38, 39). As a multi-targeted TKI inhibiting VEGFR1-3, FGFR1-4, and PDGFRα, Lenvatinib demonstrated superior efficacy over sorafenib in the REFLECT phase III trial, with a median overall survival (OS) of 13.6 months (versus 12.3 months for sorafenib) and an objective response rate (ORR) of 24.1% (compared to 9.2% for sorafenib) (5, 6, 40). However, the therapeutic potential of Lenvatinib is still frequently hindered by acquired resistance mechanisms (8, 41). One of the critical contributors to Lenvatinib resistance is the enrichment of CSCs within the tumors (42, 43).

Extensive studies have established a close link between CSCs and HCC recurrence, metastasis, and drug resistance (12, 13). CSCs are a subset of tumor cells with self-renewal capacity, multilineage differentiation potential, and high tumorigenicity. Several keys signaling, such as Wnt/β-catenin, Notch and Hedgehog pathways, have been well-documented to play vital roles in maintaining stemness properties of CSCs (15, 16). Aberrant activation of the Wnt/β-catenin pathway in malignant tumors, often due to CTNNB1 gain-of-function or APC loss-of-function mutations, leads to the upregulation of crucial target genes, such as c-MYC, Cyclin D1, and SOX9, which are involved in promoting cancer cell proliferation, survival, and sustaining CSCs stemness (17, 22, 23). Accumulating evidences suggest that acquired resistance to TKIs is associated with the enrichment of CSCs populations (42, 43). In this context, Wnt/β-catenin signaling emerges as a key contributor, not only in maintaining CSCs stemness but also in driving TKI resistance (8). The aberrant activation of this pathway helps CSCs survive and proliferate despite treatment, making it an important factor in the development of resistance to therapies like TKIs (21, 29). Given the significant role of Wnt signaling in both CSCs maintenance and TKI resistance, targeting this pathway might provide a promising therapeutic strategy. Interestingly, a recent study showed that the combination of Lenvatinib with the CDK6 inhibitor palbociclib can overcome cell resistance to Lenvatinib by blocking the Wnt/β-catenin pathway (31). This approach highlights the potential for combination therapies to effectively target both CSCs stemness and the underlying mechanisms of drug resistance.

In this study, we identified C504244 as a novel compound that inhibits Wnt/β-catenin signaling and effectively suppresses malignant phenotypes of HCC cells. Functionally, C504244 treatment led to reduced cell proliferation, migration, and invasion, along with a marked decrease in CSCs-associated features (44). Mechanistically, we found that C504244 suppressed Wnt/β-catenin pathway by inhibiting the formation of β-catenin/TCF4 complex, thereby weakening the binding of such complex to target genes’ promoter and inhibiting downstream genes’ expression. It is worth noticing that although our data indicate reduced interaction between β-catenin and TCF4 upon C504244 treatment, we do not yet have direct evidence that C504244 physically disrupts the formation of the β-catenin/TCF4 complex. Importantly, our results also suggest that the inhibitory function of C504244 is largely dependent on β-catenin signaling, as β-catenin knockdown did not further enhance the anti-tumor effects of C504244. Further studies are needed to clarify the precise mechanism by which C504244 interferes with the β-catenin/TCF4 transcriptional complex, including whether it directly disrupts their interaction interface, induces conformational changes, or acts through other mechanisms.

Given the limited efficacy of Lenvatinib monotherapy, combination therapies are actively being explored (3, 4). Previous studies have reported that Lenvatinib combined with PD-1 inhibitors (e.g., pembrolizumab) benefits selected patients with high PD-L1 expression (7, 45). Additionally, Lenvatinib in combination with suberoylanilide hydroxamic acid (SAHA), a histone deacetylase inhibitor, has been shown to enhance therapeutic outcomes (35). For EGFR-positive HCC patients, the combination of Lenvatinib and the EGFR inhibitor gefitinib significantly improves ORR (46).

In our patient-derived organoid database (33), we observed that Wnt signaling is significantly upregulated in Lenvatinib-resistant HCC samples, suggesting that Wnt activation might contribute to Lenvatinib resistance. Thus, targeting Wnt signaling holds the possibility to overcome Lenvatinib resistance and improve therapeutic efficacy. Interestingly, both *in vitro* and *in vivo* studies revealed that C504244 enhances Lenvatinib sensitivity in resistant HCC cell lines. These findings suggest that C504244 not only suppresses CSCs stemness and malignant phenotypes in HCC cells but also potentiates Lenvatinib’s therapeutic efficacy by counteracting Wnt-driven resistance mechanisms.

Compared to these approaches, C504244 offers a unique mechanism that integrates CSCs-targeting and anti-angiogenesis strategies, potentially overcoming the limitations of existing combination regimens. However, further validation in patient-derived organoids or humanized patient-derived xenograft models is necessary to translate these findings into clinical applications. Future studies should further investigate whether Wnt activation can be used as a predictive biomarker for Lenvatinib resistance and whether C504244’s efficacy extends to a broader range of resistant models.

In conclusion, C504244, a novel compound that suppresses CSCs stemness, offers a potential strategy to overcome Lenvatinib resistance in HCC. Its synergistic effect with Lenvatinib enhances treatment efficacy in resistant HCC models. Given Lenvatinib’s current clinical positioning, this combination therapy may help bridge the gap between CSCs-targeting and anti-angiogenesis strategies, providing a new avenue for improving patient outcomes. However, further studies are needed to optimize its pharmacokinetic properties, validate its efficacy in patient-derived models, and explore its clinical translation potentials.

## Data Availability

The datasets presented in this study can be found in online repositories. The names of the repository/repositories and accession number(s) can be found in the article/**Supplementary Material**.

## Supplementary material

Supplementary Figure 1 NMR, HRESIMS, and HPLC characterization of compound C504244. **(A)** ^1^H NMR Spectrum (400MHz) of compound 504244 in DMSO-d6. **(B)** ^13^C NMR Spectrum (100MHz) of compound 504244 in DMSO-d6. **(C)** HRESIMS Spectrum of compound 504244. **(D)** HPLC trace of compound 504244.

Supplementary Figure 2 Identification of C504244 as a potent inhibitor of tumor sphere formation. Representative images of spheroids formed under each treatment condition are shown. Compound 31 exhibited the strongest inhibitory effect.

Supplementary Figure 3 Cytotoxicity of C504244 in normal human cells. The IC_50_ values of C504244 in cells were determined using cell viability assay. Huh7, SK-Hep1, HUVEC, HDF, WI-38, and PBMC cells were treated with C504244 at indicated dosages for 48h, and cell viability was measured using the CCK-8 assay.

Supplementary Figure 4 C504244 functions mainly via suppressing β-catenin signaling in HCC cells. **(A)** Huh7 cells were transfected with siRNA targeting β-catenin (20 nM) or negative control, and treated with DMSO or C504244 (2 µM) for 48 hours. GAPDH was detected as the loading control. **(B)** ALDH activity was analyzed by flow cytometry, and the percentage of ALDH+ cells was quantified. **(C)** Cell proliferation was assessed by cell growth curve analysis in Huh7 cells. Cell numbers were detected every two days and monitored until day 9 after plating. Relative cell growth was normalized to day 1. **(D)** Cell migration was evaluated by wound healing assay in Huh7 cells. Images were captured at 0- and 24-hours post-scratching. Scale bar = 100 µm. All statistical analyses were performed using Student’s t-test, with significance indicated as **p* < 0.05.

Supplementary Figure 5 Canonical Wnt signaling correlates with stemness in HCC. **(A)** GSVA scores of the canonical Wnt signaling pathway were compared between tumor and adjacent liver tissue derived organoids and corresponding primary tissues. **(B)** A positive correlation was observed between canonical Wnt signaling activity and embryonic stem cell-like signatures in HCC organoids.

Supplementary Figure 6 Synergistic effects of Lenvatinib and C504244 in SK-Hep1 cells. **(A-C)** SK-Hep1 cells treated with DMSO, Lenvatinib, C504244, or the combination of both drugs were applied for colony formation **(A)**, migration **(B)**, and wound healing **(C)** assays.

Supplementary Figure 7 Synergistic effect of Lenvatinib and C504244 in Huh7 cells. **(A)** Huh7 cells treated with DMSO, Lenvatinib or C504244 were collected for western blotting analysis. GAPDH was detected as loading control. **(B)** SK-Hep1 and Hep1–6 cells treated with C504244 for 48 hours at indicated concentrations were collected for qPCRs analysis.

Supplementary Figure 8 Synergistic effect of Lenvatinib and C504244 in Hep1–6 cells. **(A)** Synergy map of Hep1–6 cells treated with indicated concentrations of C504244 and Lenvatinib. **(B)** Cell proliferation was assessed by cell growth curve analysis in Hep1–6 cells treated with DMSO, Lenvatinib (4 µM), C504244 (4 µM), or the combination. Cell numbers were counted every two days and monitored until day 9. Relative cell growth was normalized to day 0. **(C)** Colony formation assay was performed on Hep1–6 cells treated with DMSO, Lenvatinib (4 µM), C504244 (4 µM), or the combination for 14 days. Colony formation efficiency was quantified by counting colonies and expressing the results as a percentage relative to the vehicle control. **(D)** Cell migration was evaluated using a migration assay, where Hep1–6 cells were treated with DMSO, Lenvatinib, C504244, or the combination for 24 hours. Migrated cells were stained with crystal violet, and the relative number of cells that migrated through the membrane was quantified. Scale bar = 100 µm. All statistical analyses were performed using Student’s *t*-test, with significance indicated as **p* < 0.05.

Supplementary Figure 9 Evaluation of C504244 and combination treatment on organ histology. Histological analysis of major organs (heart, kidney, liver, spleen, and lung) from Hep1–6 tumor-bearing nude mice after treatment with vehicle, Lenvatinib, C504244, or the combination for 40 days. Representative H&E-stained sections from each group (n = 6) are shown. No evident tissue damage, inflammation, or necrosis was observed in any treatment group, indicating no overt systemic toxicity. Scale bar = 100 µm.
