# Supplementary figures and images for: Diaryl pyrimidine guanidine suppresses hepatocellular carcinoma cell stemness by targeting β-catenin signaling
Source: Front Oncol. 2025 Sep 1;15:1641979. doi: 10.3389/fonc.2025.1641979 (PMC12434761; doi:10.3389/fonc.2025.1641979)

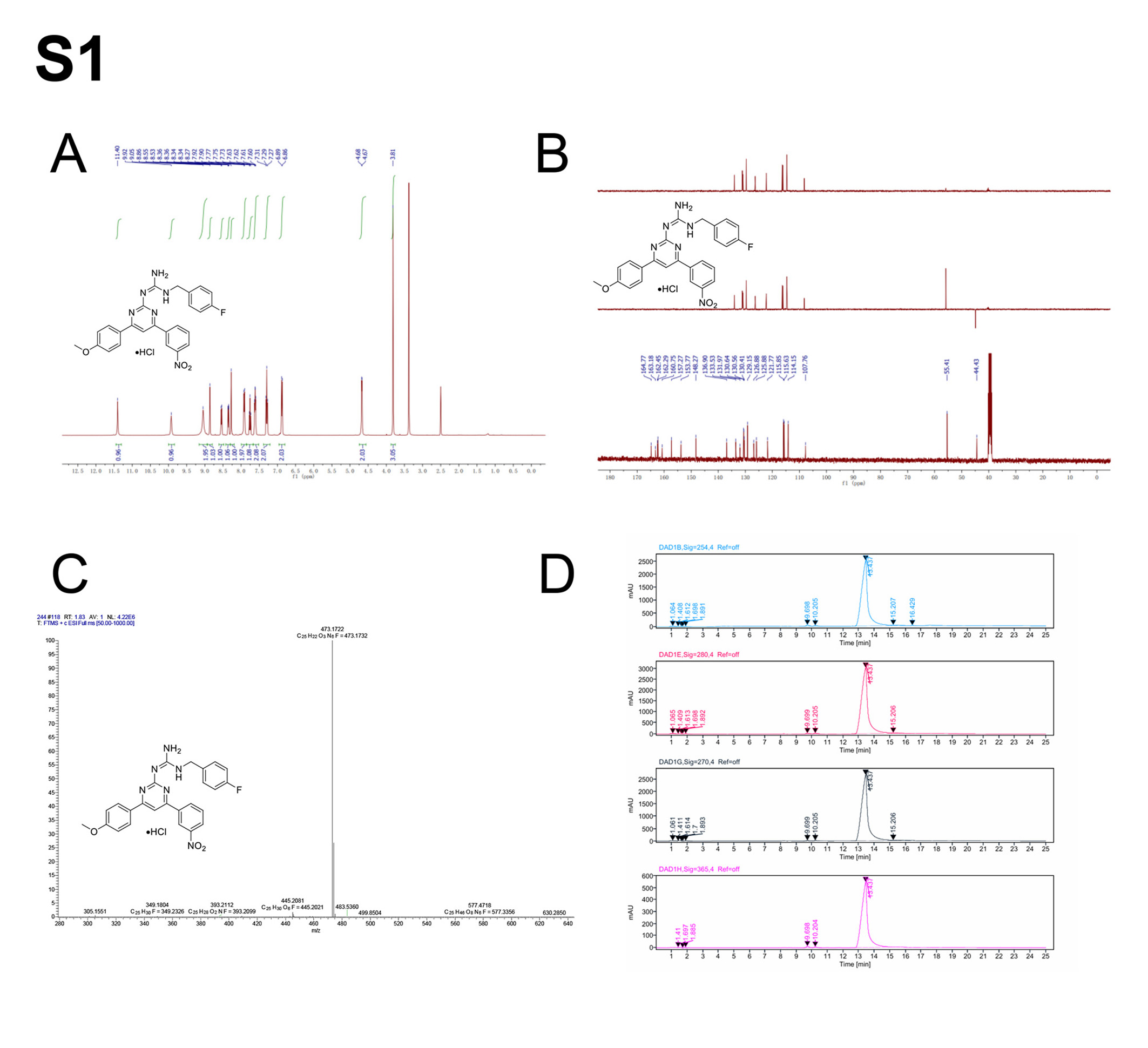

Supplement: Supplementary Figure 1 — NMR, HRESIMS, and HPLC characterization of compound C504244. (A) 1H NMR Spectrum (400MHz) of compound 504244 in DMSO-d6. (B) 13C NMR Spectrum (100MHz) of compound 504244 in DMSO-d6. (C) HRESIMS Spectrum of compound 504244. (D) HPLC trace of compound 504244. [file DataSheet1.zip › Supplementary-figure1-9/S1.jpg]

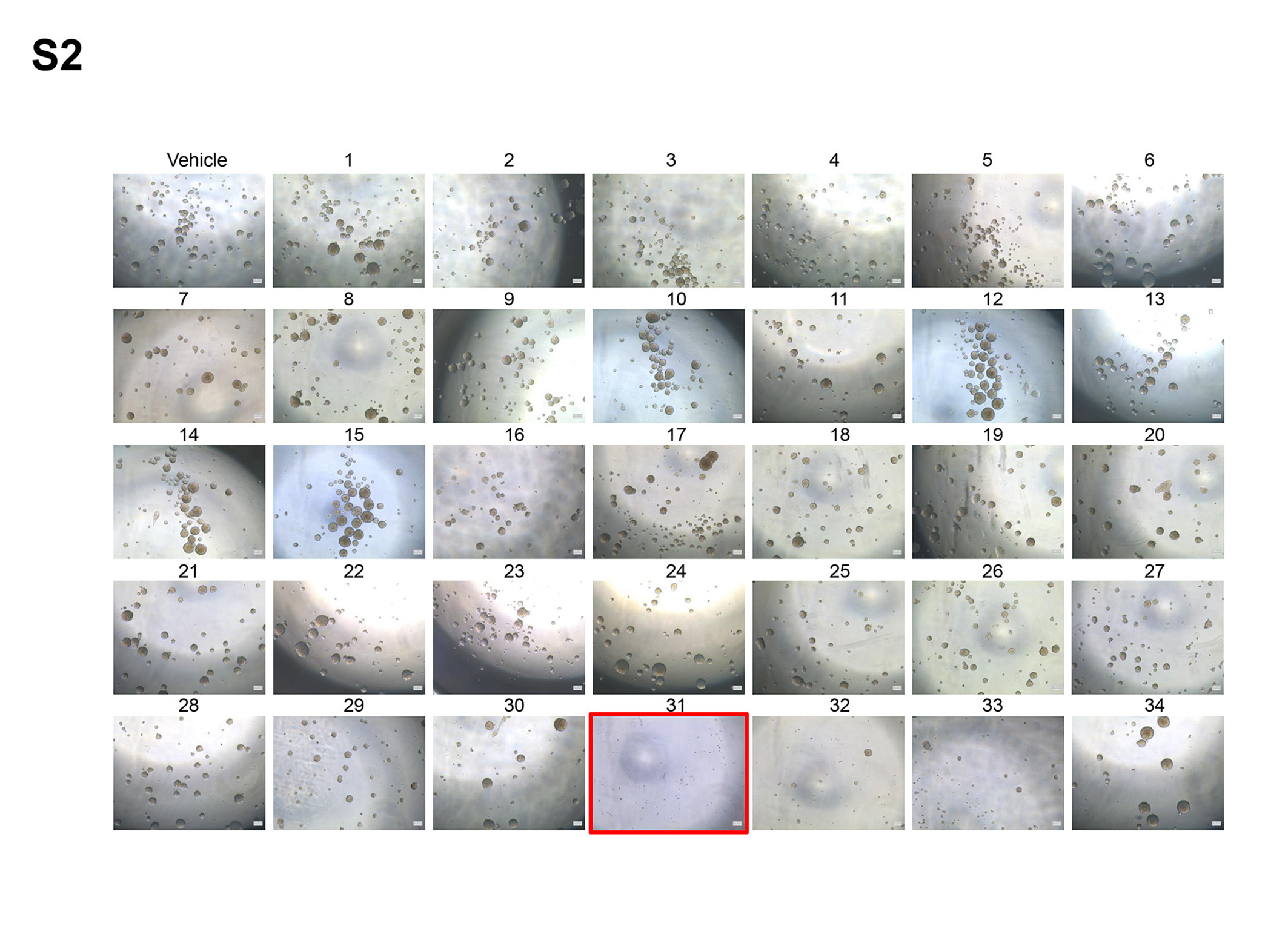

Supplement: Supplementary Figure 1 — NMR, HRESIMS, and HPLC characterization of compound C504244. (A) 1H NMR Spectrum (400MHz) of compound 504244 in DMSO-d6. (B) 13C NMR Spectrum (100MHz) of compound 504244 in DMSO-d6. (C) HRESIMS Spectrum of compound 504244. (D) HPLC trace of compound 504244. [file DataSheet1.zip › Supplementary-figure1-9/S2.jpg]

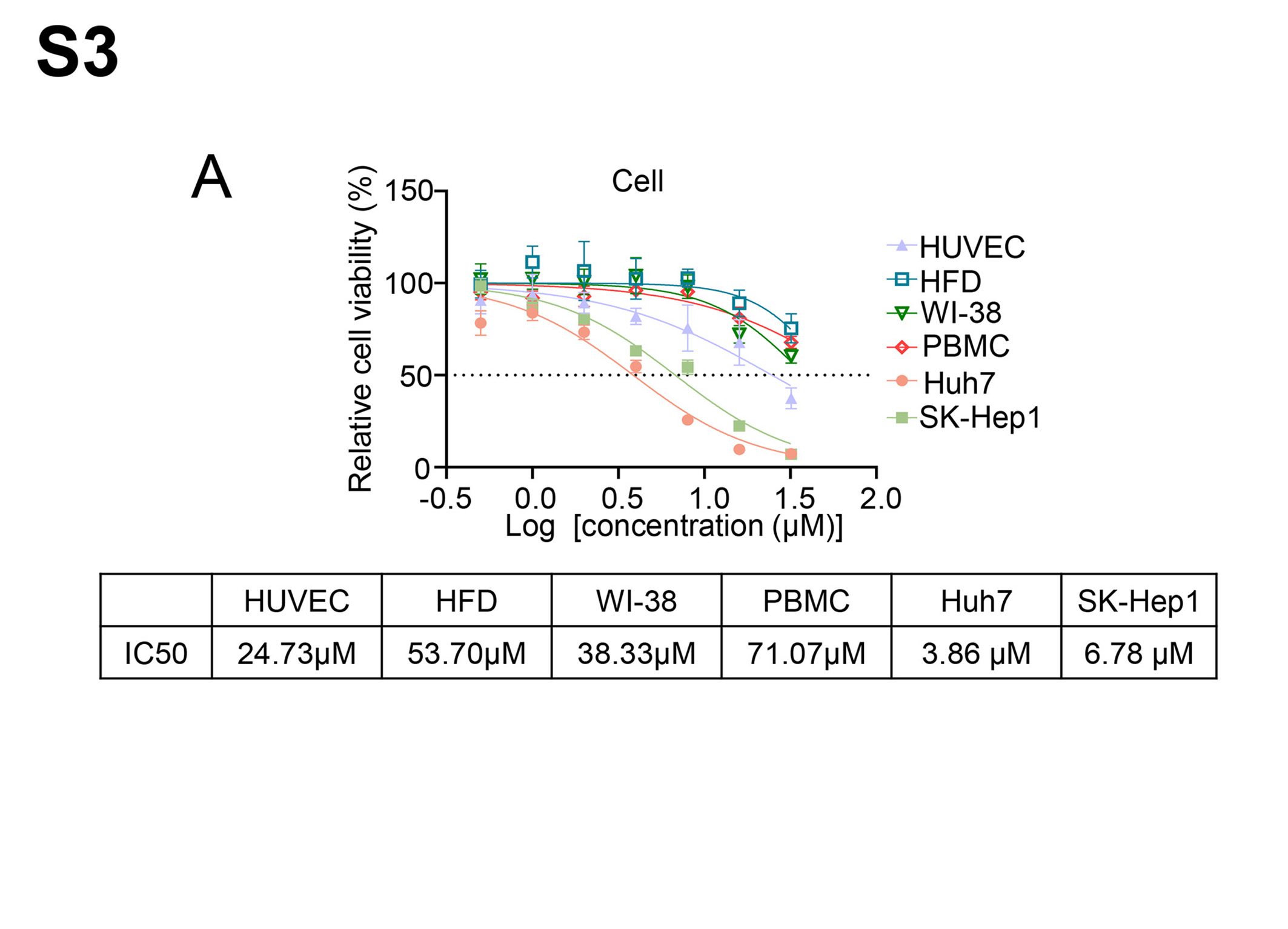

Supplement: Supplementary Figure 1 — NMR, HRESIMS, and HPLC characterization of compound C504244. (A) 1H NMR Spectrum (400MHz) of compound 504244 in DMSO-d6. (B) 13C NMR Spectrum (100MHz) of compound 504244 in DMSO-d6. (C) HRESIMS Spectrum of compound 504244. (D) HPLC trace of compound 504244. [file DataSheet1.zip › Supplementary-figure1-9/S3.jpg]

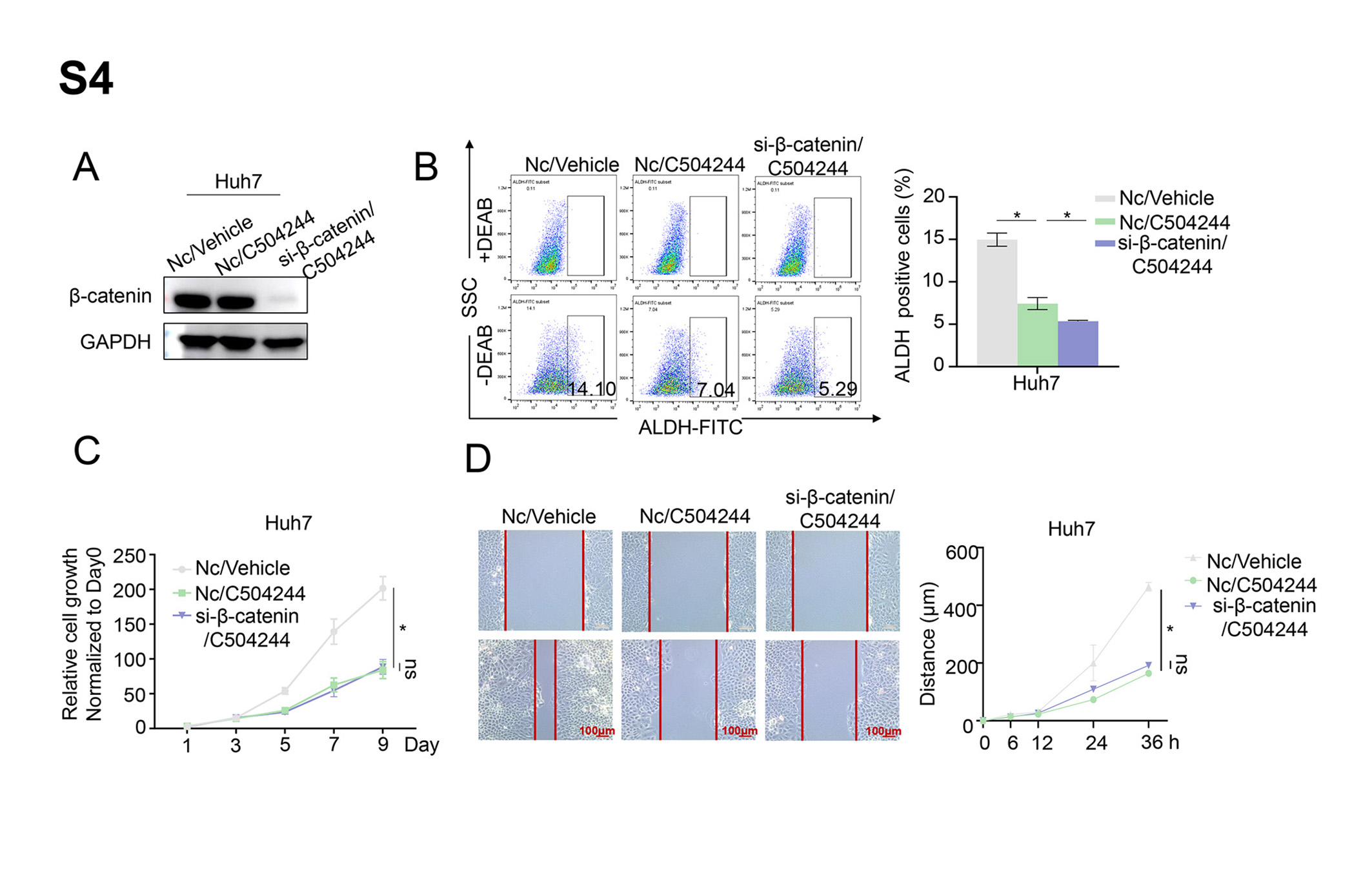

Supplement: Supplementary Figure 1 — NMR, HRESIMS, and HPLC characterization of compound C504244. (A) 1H NMR Spectrum (400MHz) of compound 504244 in DMSO-d6. (B) 13C NMR Spectrum (100MHz) of compound 504244 in DMSO-d6. (C) HRESIMS Spectrum of compound 504244. (D) HPLC trace of compound 504244. [file DataSheet1.zip › Supplementary-figure1-9/S4.jpg]

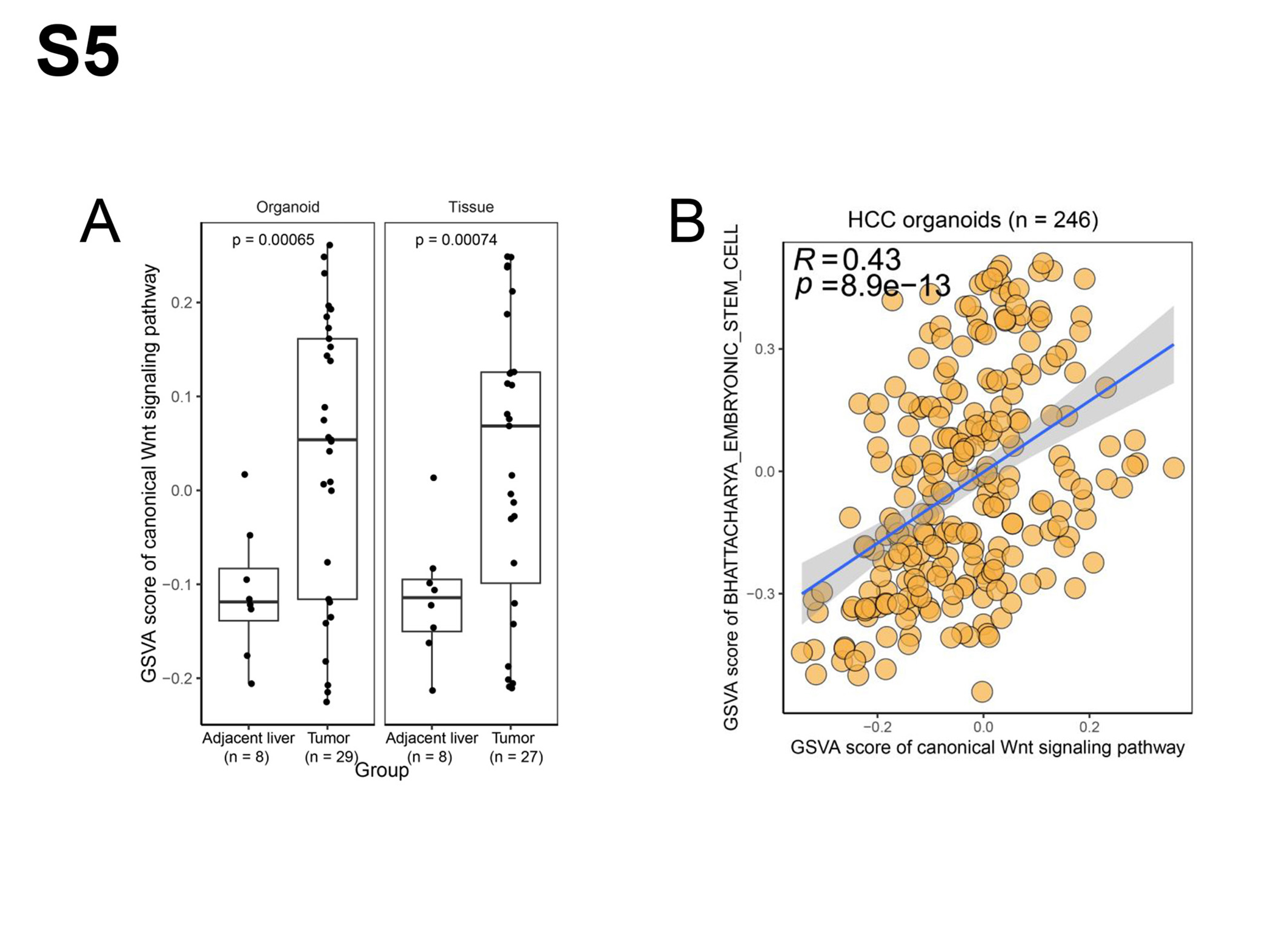

Supplement: Supplementary Figure 1 — NMR, HRESIMS, and HPLC characterization of compound C504244. (A) 1H NMR Spectrum (400MHz) of compound 504244 in DMSO-d6. (B) 13C NMR Spectrum (100MHz) of compound 504244 in DMSO-d6. (C) HRESIMS Spectrum of compound 504244. (D) HPLC trace of compound 504244. [file DataSheet1.zip › Supplementary-figure1-9/S5.jpg]

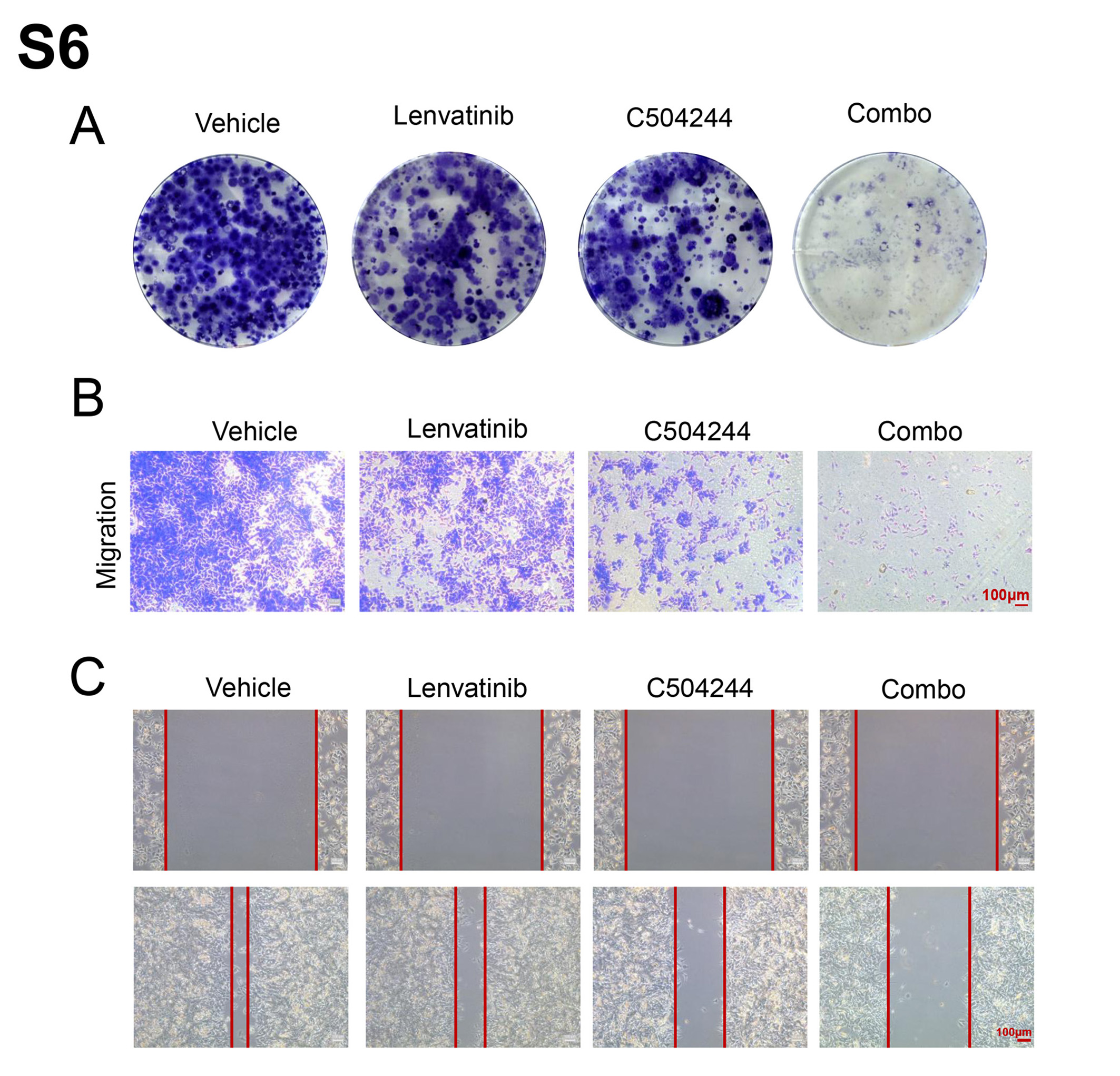

Supplement: Supplementary Figure 1 — NMR, HRESIMS, and HPLC characterization of compound C504244. (A) 1H NMR Spectrum (400MHz) of compound 504244 in DMSO-d6. (B) 13C NMR Spectrum (100MHz) of compound 504244 in DMSO-d6. (C) HRESIMS Spectrum of compound 504244. (D) HPLC trace of compound 504244. [file DataSheet1.zip › Supplementary-figure1-9/S6.jpg]

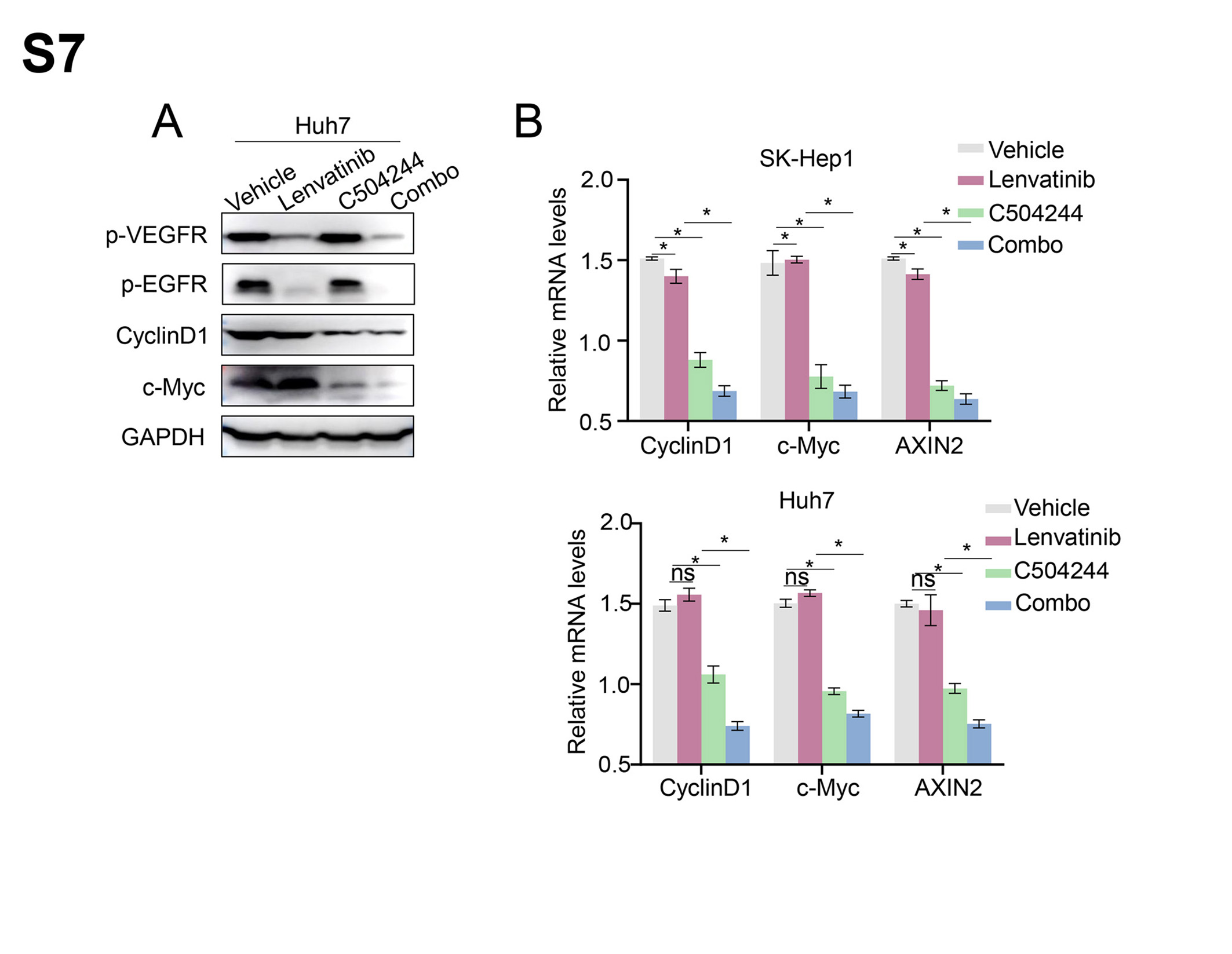

Supplement: Supplementary Figure 1 — NMR, HRESIMS, and HPLC characterization of compound C504244. (A) 1H NMR Spectrum (400MHz) of compound 504244 in DMSO-d6. (B) 13C NMR Spectrum (100MHz) of compound 504244 in DMSO-d6. (C) HRESIMS Spectrum of compound 504244. (D) HPLC trace of compound 504244. [file DataSheet1.zip › Supplementary-figure1-9/S7.jpg]

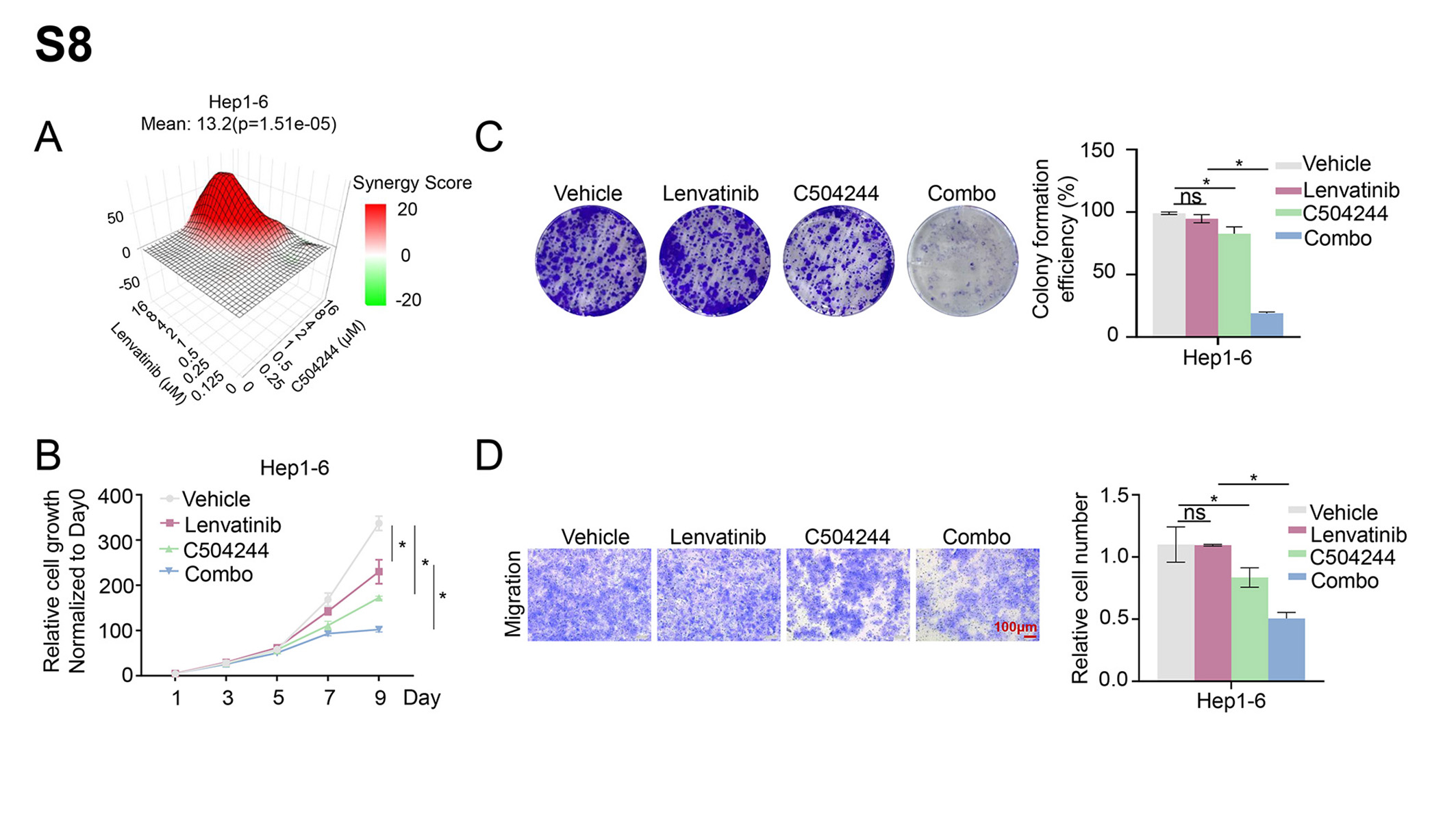

Supplement: Supplementary Figure 1 — NMR, HRESIMS, and HPLC characterization of compound C504244. (A) 1H NMR Spectrum (400MHz) of compound 504244 in DMSO-d6. (B) 13C NMR Spectrum (100MHz) of compound 504244 in DMSO-d6. (C) HRESIMS Spectrum of compound 504244. (D) HPLC trace of compound 504244. [file DataSheet1.zip › Supplementary-figure1-9/S8.jpg]

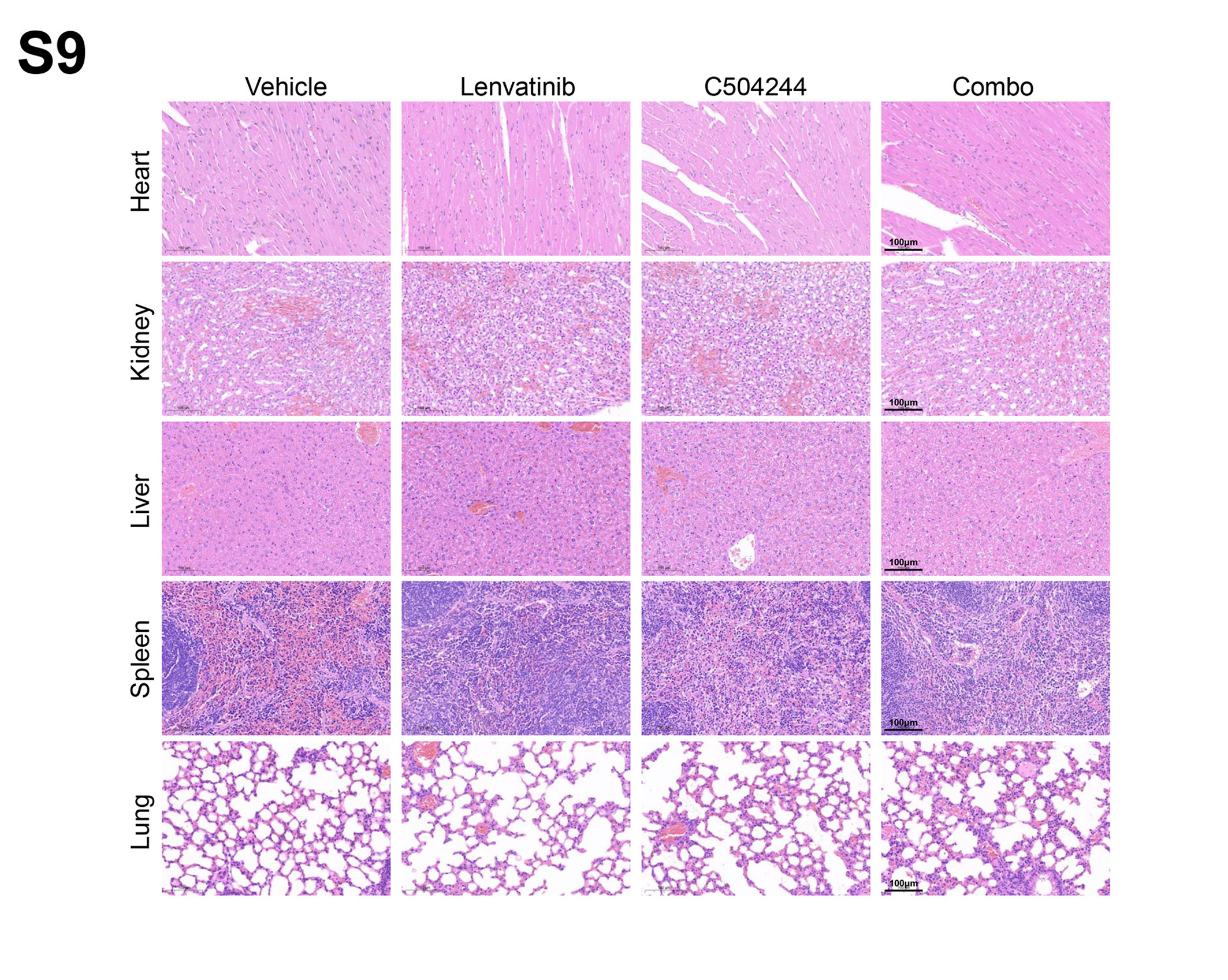

Supplement: Supplementary Figure 1 — NMR, HRESIMS, and HPLC characterization of compound C504244. (A) 1H NMR Spectrum (400MHz) of compound 504244 in DMSO-d6. (B) 13C NMR Spectrum (100MHz) of compound 504244 in DMSO-d6. (C) HRESIMS Spectrum of compound 504244. (D) HPLC trace of compound 504244. [file DataSheet1.zip › Supplementary-figure1-9/S9.jpg]
