# Supplementary material for: Diaryl pyrimidine guanidine suppresses hepatocellular carcinoma cell stemness by targeting β-catenin signaling
Source: Front Oncol. 2025 Sep 1;15:1641979. doi: 10.3389/fonc.2025.1641979 (PMC12434761; doi:10.3389/fonc.2025.1641979)
Supplement: Supplementary file 2 [file Table1.docx]

**Supplementary Table1**. Sequences of the DNA primers

| Name | Sequence (5’-3’) |
| --- | --- |
| ALDH1 | Forward: CGGGAAAAGCAATCTGAAGAGGG  Reverse: GATGCGGCTATACAACACTGGC |
| OCT4 | Forward: GAAGGATGTGGTCCGAGTGT  Reverse: GTGAAGTGAGGGCTCCCATA |
| Nanog | Forward: TTCCTTCCTCCATGGATCTG  Reverse: ATCTGCTGGAGGCTGAGGTA |
| GAPDH | Forward: TGACAACGAATTTGGCTACA  Reverse: GTGGTCCAGGGGTCTTACTC |
| SOX9 | Forward: GGAGATGAAATCTGTTCTGGGAATG  Reverse: TTGAAGGTTAACTGCTGGTGTTCTG |
| SOX2 | Forward: GCACAACTCGGAGATCAGCA  Reverse: TTCATGTGCGCGTAACTGTC |
| c-Myc | Forward: AGTACTGCTACGGAGGAGCA  Reverse: GCGGAGATTAGCGAGAGAGG |
| Cyclin D1 | Forward: GAGGTGTGTTTCTCCCGGTT  Reverse: GAAAATTCCAGCAGCAGCCC |

**Supplementary Table2. Structural Characterization Data of C504244**

| Experiment | Result |
| --- | --- |
| Melting point (M.p.) | 172-173 ℃ |
| ^1^H NMR (400 MHz, DMSO-*d*) | \|  \| \| --- \|  \| δ (ppm): 11.40 (s, 1H), 9.92 (s, 1H), 9.05 (s, 2H), 8.86 (s, 1H, aromatic), 8.54 (d, J = 7.8 Hz, 1H, aromatic), 8.35 (dd, J = 8.2, 2.2 Hz, 1H, aromatic), 8.27 (s, 1H, aromatic), 7.91 (d, J = 8.5 Hz, 2H, aromatic), 7.75 (t, J = 8.0 Hz, 1H, aromatic), 7.61 (dd, J = 8.3, 5.5 Hz, 2H, aromatic), 7.29 (t, J = 8.6 Hz, 2H, aromatic), 6.87 (d, J = 8.4 Hz, 2H, aromatic), 4.67 (d, J = 5.2 Hz, 2H, N-CH₂), 3.81 (s, 3H, OCH₃) \| \| --- \| |
| ¹³C NMR (100 MHz, DMSO-d₆) | δ (ppm): 164.8, 163.2 (d, ^1^*J*_CF_ = 244.2 Hz), 162.4, 162.3, 157.3, 153.8, 148.3, 136.9, 133.5, 132.0 (d, ^4^*J*_CF_ = 3.2 Hz), 130.6, (d, ^3^*J*_CF_ = 8.5 Hz), 130.4, 129.1, 126.9, 125.9, 121.8, 115.8 (d, ^2^*J*_CF_ = 21.7 Hz), 114.1, 107.8, 55.4, 44.4. |
| HRMS-ESI | *m/z* calced for C_25_H_22_N_6_O_3_F [M+H]^+^ 473.1732, found 473.1722. |

**Supplementary Table3. Area percent report of compound 504244**

Signal, 254 nm

| Number | Retention time (min) | Area | Area% |
| --- | --- | --- | --- |
| 1 | 1.064 | 358.8981 | 0.4970 |
| 2 | 1.408 | 9.5883 | 0.0133 |
| 3 | 1.612 | 23.4539 | 0.0325 |
| 4 | 1.698 | 10.9979 | 0.0152 |
| 5 | 1.891 | 114.1901 | 0.1581 |
| 6 | 9.698 | 534.7437 | 0.7406 |
| 7 | 10.205 | 276.1551 | 0.3824 |
| 8 | 13.437 | 70130.5240 | 97.1227 |
| 9 | 15.207 | 677.1962 | 0.9378 |
| 10 | 16.429 | 72.4091 | 0.1003 |
| Total |  | 72208.1565 | 100.0 |

Signal, 270 nm

| Number | Retention time (min) | Area | Area% |
| --- | --- | --- | --- |
| 1 | 1.061 | 341.2590 | 0.4428 |
| 2 | 1.411 | 7.3819 | 0.0096 |
| 3 | 1.614 | 21.1746 | 0.0275 |
| 4 | 1.700 | 10.7142 | 0.0139 |
| 5 | 1.893 | 139.3784 | 0.1808 |
| 6 | 9.699 | 346.2073 | 0.4492 |
| 7 | 10.205 | 364.5132 | 0.4729 |
| 8 | 13.437 | 75128.1536 | 97.4772 |
| 9 | 15.206 | 713.7269 | 0.9260 |
| Total |  | 77072.5093 | 100.0 |

Signal, 280 nm

| Number | Retention time (min) | Area | Area% |
| --- | --- | --- | --- |
| 1 | 1.065 | 296.3028 | 0.3317 |
| 2 | 1.409 | 7.7928 | 0.0087 |
| 3 | 1.613 | 20.5433 | 0.0230 |
| 4 | 1.698 | 9.6837 | 0.0108 |
| 5 | 1.892 | 132.2279 | 0.1480 |
| 6 | 9.699 | 326.4777 | 0.3655 |
| 7 | 10.205 | 389.5680 | 0.4361 |
| 8 | 13.437 | 87287.4898 | 97.7202 |
| 9 | 15.206 | 853.8555 | 0.9559 |
| Total |  | 89323.9415 | 100.0 |

Signal, 365 nm

| Number | Retention time (min) | Area | Area% |
| --- | --- | --- | --- |
| 1 | 1.410 | 7.1082 | 0.0470 |
| 2 | 1.697 | 6.6444 | 0.0440 |
| 3 | 1.885 | 71.5677 | 0.4735 |
| 4 | 9.698 | 143.2659 | 0.9479 |
| 5 | 10.204 | 92.6935 | 0.6133 |
| 6 | 13.437 | 14792.0981 | 97.8742 |
| Total |  | 15113.3778 | 100.0 |

**Supplementary Table4. Prediction of physicochemical properties of compound 504244 ^a^**

| Compound | cLogP | TPSA | MW | HBA | HBD |
| --- | --- | --- | --- | --- | --- |
| Standard | < 5 | < 140 | < 500 | < 10 | < 5 |
| **504244** | 3.77 | 128.56 | 472 | 9 | 3 |

^a^cLogP: calculated logarithm of the octanol-water partition coefficient; TPSA: topological polar surface area; MW: molecular weight; HBA: hydrogen-bond acceptor atoms. HBD: hydrogen-bond donor atoms; Lipinski Rules: MW ≤ 500; logP ≤ 5; HBA ≤ 10; HBD ≤ 5, If two properties are out of range, a poor absorption or permeability is possible, one is acceptable.
